# Expression and Functional Analysis of WRKY Transcription Factors in Chinese Wild Hazel, *Corylus heterophylla* Fisch

**DOI:** 10.1371/journal.pone.0135315

**Published:** 2015-08-13

**Authors:** Tian-Tian Zhao, Jin Zhang, Li-Song Liang, Qing-Hua Ma, Xin Chen, Jian-Wei Zong, Gui-Xi Wang

**Affiliations:** 1 State Key Laboratory of Tree Genetics and Breeding, Research Institute of Forestry, Chinese Academy of Forestry, Beijing, China; 2 Shandong Institute of Pomology, Shandong Provincial Key Laboratory of Fruit Tree Biotechnology Breeding, Tai’an, Shandong, China; Institute of Genetics and Developmental Biology, Chinese Academy of Sciences, CHINA

## Abstract

Plant WRKY transcription factors are known to regulate various biotic and abiotic stress responses. In this study we identified a total of 30 putative WRKY unigenes in a transcriptome dataset of the Chinese wild Hazel, *Corylus heterophylla*, a species that is noted for its cold tolerance. Thirteen full-length of these *ChWRKY* genes were cloned and found to encode complete protein sequences, and they were divided into three groups, based on the number of WRKY domains and the pattern of zinc finger structures. Representatives of each of the groups, Unigene25835 (group I), Unigene37641 (group II) and Unigene20441 (group III), were transiently expressed as fusion proteins with yellow fluorescent fusion protein in *Nicotiana benthamiana*, where they were observed to accumulate in the nucleus, in accordance with their predicted roles as transcriptional activators. An analysis of the expression patterns of all 30 *WRKY* genes revealed differences in transcript abundance profiles following exposure to cold, drought and high salinity conditions. Among the stress-inducible genes, 23 were up-regulated by all three abiotic stresses and the WRKY genes collectively exhibited four different patterns of expression in flower buds during the overwintering period from November to April. The organ/tissue related expression analysis showed that 18 WRKY genes were highly expressed in stem but only 2 (*Unigene9262* and *Unigene43101*) were greatest in male anthotaxies. The expression of *Unigene37641*, a member of the group II *WRKY* genes, was substantially up-regulated by cold, drought and salinity treatments, and its overexpression in *Arabidopsis thaliana* resulted in better seedling growth, compared with wild type plants, under cold treatment conditions. The transgenic lines also had exhibited higher soluble protein content, superoxide dismutase and peroxidase activiety and lower levels of malondialdehyde, which collectively suggets that *Unigene37641* expression promotes cold tolerance.

## Introduction

Members of the *Corylus* genus, commonly referred to as hazel, are economically and ecologically import plants in many parts of the world. Hazelnut kernels are rich in unsaturated fats, vitamin E, arginine, glutamic acid, and aspartic acid [1–3], and the nuts are incorporated in butters, pastes, confectionary spreads, flours, and are also widely used in the chocolate industry [4, 5]. In addition, the compound taxol (paclitaxel), a drug that is used in cancer therapy, is found in many hazelnut species [6, 7]. The major countries producing hazel for commercial purposes are Turkey, Italy, America, Azerbaijan, Georgia, and China [8–10], and currently more than 70% of the world’s production originates from the Black Sea region. More than 4 million acres of natural hazel grow in the northeastern and northwestern regions of China, with an annual yield of more than 23,000 tons [11–12].

The hazel species *C*. *heterophylla* Fisch is widely distributed in northern China where, for centuries, its nuts have been harvested for oil extraction and as a food source [3]. It is an economically important species, especially in the northeast region, and its production accounts for nearly three quarters of the total output of the Chinese domestic market [13]. In addition to its economic value, *C*. *heterophylla* also plays a key role in water and soil conservation and in the ecological balance of certain types of forests [14]. Some of the qualities that make *C*. *heterophylla* especially attractive as a hazelnut crop species include high productivity, early maturity, resistance to Eastern Filbert Blight (EFB), a fungus that is found in the common hazel, *C*. *avellana* [15, 16] and cold hardiness. Indeed, *C*. *heterophylla* has been shown to tolerate temperatures as low as -48°C, while *C*. *avellana* cannot [3, 17]. These desirable traits also make it a potentially important genetic resource for selection and breeding [18], and a *C*. *heterophylla* × *C*. *avellana* interspecific crossing project has been initiated in China and Korea [3].

The marked cold stress tolerance of *C*. *heterophylla* has provoked interest in identifying the key genes involved in the associated cold stress responses and the underlying signaling pathways [19]. Abiotic factors such as cold, drought, and high salinity, can cause severe damage in plants, resulting in major losses in crop yield and quality worldwide [20, 21]. In order to survive, plants to such abiotic stresses include numerous and complex biochemical and physiological changes [22]. The perception, signal transduction and molecular response mechanism of the external stimuli has been analyzed at the transcriptional level in many plant species [23, 24]. In the previous study, we used high resolution RNA-sequencing (RNA-seq) technology to elucidate the mechanisms of cold tolerance used by *C*. *heterophylla*, through a transcriptional profiling of four stages of floral buds, including non-cold acclimation (NA), cold acclimation (CA), midwinter (MW), and de-acclimation (DA) samples [25]. RNA-seq analysis has already proved useful for studies of *C*. *avellana* and *C*. *mandshurica* [2, 9]. We focused in particular on the transcriptional regulation of the cold stress responses and the role of transcription factors.

One of the largest families of transcription factors in plants is the WRKY family [26–28], members of which are known to modulate stress response [29–31]. A defining feature of this family, the WRKY domain, is a highly conserved stretch of approximately 60 amino acids with the highly conserved amino acid sequence WRKYGQK at the N-terminus, as well as the zinc finger structures C_2_H_2_ (CX_4–5_CX_22–23_HXH) or C_2_HC (CX_7_CX_23_HXC) at the C-terminus [32–35]. WRKY proteins can be classified into three groups, depending on the number of WRKY domains and the pattern of the zinc finger structures [27, 36]. In general, group I contains two WRKY domains, whereas the other two groups have only one domain. Group III contains a C_2_HC zinc finger motif that is distinct from the C_2_H_2_ motif present in group II [32]. To date, WRKY proteins have been shown to act as activators or repressors of developmental processes [27], such as trichome development [37], senescence [38, 39], embryogenesis [40], and seed development [41]. In addition, they have been shown to play a role in defense against biotic and abiotic stresses, including bacterial [42] and viral pathogens [43], wounding [44] drought, high salinity, cold and freezing [45]. However, the role of WRKY transcription factors in response to abiotic stresses is less well understood than the association with biotic stress factors [36, 46].

Since the first description of a WRKY protein, SPF1 from sweet potato (*Ipomoea batatas*) [47], large numbers of WRKY proteins have been identified from numerous plant species [48–55]. However, to date no WRKY transcription factor has been reported for any other *Corylus* species. In this study, we identified 30 members of the *C*. *heterophylla* WRKY transcription factor family, based on a transcriptome analysis of floral buds. We evaluated their expression in various tissues/organs under normal growing conditions and also following exposure to different abiotic stresses, including cold, drought and high salinity. In addition, we determined the subcellular localization of three members of the WRKY family and performed a functional assessment of one *WRKY* gene, *Unigene37641*, by overexpression in transgenic *Arabidopsis thaliana* plants.

## Materials and Methods

### Ethics statement

All necessary permits for field sampling were obtained from the local forestry department and Chinese Academy of Forestry.

### Plant material and stress treatments

*C*. *heterophylla* was obtained from Weichang, Chengde, China (41°58′ N, 117°40′ E). Floral buds were collected on the first day of each month from November 2011 to March 2012. For organ/tissue-specific expression analysis, the stem and male anthotaxy was also collected in December. *C*. *heterophylla* plants were grown in pots containing sand and turf peat (1:2 v/v) in controlled environment growth chambers (16 h light/8 h dark cycle; 100 μmol·m^−2^·s^−1^ light intensity; 25°C) for two months. For drought, salinity, and cold stress treatments, seedlings were subjected to 25% (w/v) Polyethylene glycol (PEG) 6000, 400 mM NaCl, and 4°C conditions, respectively, as previously described by Wang *et al* [56]. Untreated seedlings were grown under the same environmental. Leaf samples were collected at 2 h, 4 h, 8 h and 24 h after treatment, frozen in liquid nitrogen and stored at −80°C prior to RNA extraction.

### Cloning and sequence analysis of *ChWRKY* genes

Total RNA from *C*. *heterophylla* leaves, floral buds, stems and male anthotaxies were extracted using an RNA Extract Kit (Aidlab, Beijing, China). Based on the functional annotation of the *C*. *heterophylla* transcriptome (National Center for Biotechnology Information SRA database, accession number: SRX529300) [25], the assembled sequences were subjected to BLASTX (http://blast.ncbi.nlm.nih.gov/) analysis against the *A*. *thaliana* protein database at NCBI. Based on this analysis, a total of 30 candidate sequences containing WRKY domains were selected with an *E*-value less than 10^−5^. Primers for RACE were designed based on the WRKY sequences (S1 Table) and the RACE was conducted using the BD SMART RACE cDNA Amplification Kit (Clontech, Mountain View, USA) according to the manufacturer's instructions [57]. The products from the RACE PCR were ligated into the pMD-18T vector (TaKaRa, Dalian, China), which was transformed into competent *Escherichia coli* DH5α cells. After blue-white colony screening, positive clones were identified, and sent to Beijing Genomics Institution (BGI) for sequencing. Sequences of the PtrWRKY and AtWRKY proteins were downloaded from the Phytozome v10.2 website (http://www.phytozome.net/poplar) and the *Arabidopsis* genome TAIR 9.0 website (http://www.Arabidopsis.org/index.jsp), respectively. The phylogenetic trees were constructed using MEGA4.1 (http://www.megasoftware.net/mega.html) by employing the maximum likelihood (ML) for full-length proteins and neighbour-joining (NJ) method for conserved WRKY domains with 1,000 bootstrap replicates [58, 59]. The alignments of conserved WRKY domains in each subclass were output using DNAman. The WRKY domains were predicted using the MEME (http://meme.nbcr.net/meme/cgi-bin/meme.cgi) [29, 60]. Protein subcellular localization was predicted by Euk-mPLoc 2.0 software (http://www.csbio.sjtu.edu.cn/bioinf/euk-multi-2/).

### Quantitative Real-time PCR (qRT-PCR) analysis

First strand cDNA was synthesized from total RNA using a First Strand Synthesis system (Invitrogen, USA) according to the manufacturer’s instructions. To analyze the expression levels of the WRKY transcription factors, qRT-PCR reactions were performed using the Bio-Rad CFX96 Real-Time PCR System (BIO-RAD, USA) and the SYBR Green qPCR Mix (Takara, Japan) [61]. qRT-PCR reactions were performed in a total volume of 20 μl and cycling conditions were 95°C for 30 s, followed by 39 cycles of 95°C/10 s, 60°C/15 s, 72°C/30 s, followed by a melting curve analysis [27]. The *ChActin* gene was used as an internal control [25] and the expression levels of *WRKY* genes were calculated using the 2^−ΔΔCt^ formula [62]. The primer pairs were designed using Primer 3 (http://bioinfo.ut.ee/primer3-0.4.0/primer3/) and listed in S2 Table. Each reaction was performed with three biological replicates.

### Subcellular localization assay

The sequences corresponding to the ORFs without stop codons of *Unigene25835*, *Unigene37641* and *Unigene20441* were inserted into the pEarlyGate101 vector (ABRC stock DB3-683) to produce *35S*::*ChWRKY-YFP* constructs using the Gateway cloning system (Invitrogen, USA). For subcellular localization analysis, transient expression of *Nicotiana benthamiana* lower leaf epidermal cells was performed as previously described [63] with some modifications. Plants were cultivated under short-day conditions (8 h light/ 16 h dark). When the *Agrobacterium* culture reached the stationary growth phase at 28°C with agitation, cells were pelleted and resuspended in infiltration buffer (100 μM acetosyringone in 10 mM MgCl_2_). Fluorescence was observed using a LSM 510 confocal laser scanning microscope (Carl Zeiss, Oberkochen, Germany).

### Generation of transgenic *A*. *thaliana* plants

The sequence corresponding to the *Unigene37641* ORF was cloned and inserted into the pBI121 vector using the primer pair: 5´-ACTAGTATGTCTGATGAACA—TAG-3´ and 5´-GGTACCTGGCTCTTGTTTAAAG-3´. The resulting pBI121- *Unigene37641* vector, which contained the CaMV 35S promoter to drive expression of *Unigene37641*, was transformed into *A*. *thaliana* using the floral dip method [64]. Semi-quantitative RT-PCR was used to determine the gene expression level in T_2_ transgenic lines. The wild type (WT) and transgenic seedlings were cultured on MS medium for 1 week, followed by the cold stress treatment (4°C for 6 h per day) for 2 weeks, as previously described by Li *et al* [57].

### Soluble protein, superoxide dismutase, peroxidase and malondialdehyde measurements

Six-week-old WT and T_2_ transgenic lines were transferred to a cold-chamber and maintained at 4°C under light for 12 h. Soluble protein content was determined by the coomassie brillant blue G-250 method [65]. Superoxide dismutase (SOD) was calculated as previously described by Giannopolitis *et al* [66] and peroxidase (POD) activity assats were performed using the guaiacol method [67]. The malondialdehyde (MDA) content was determined using the thiobarbituric acid method [29].

## Results

### Cloning and sequence analysis of *C*. *heterophylla* WRKY genes

**Fig 1:**
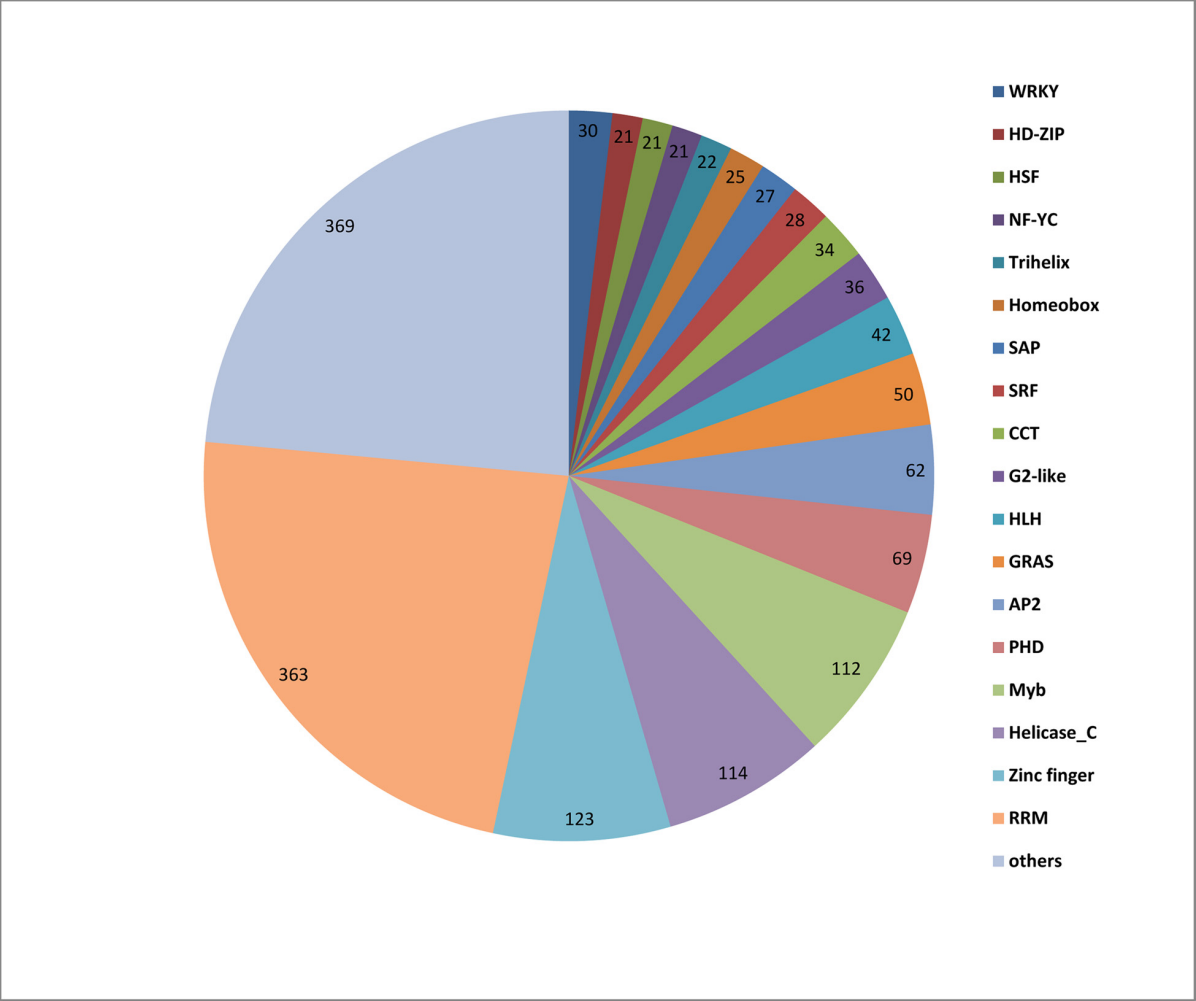
Number of unique transcripts in annotated as transcription factors and the associated transcription factor families in the *C*. *heterophylla* Fisch transcriptome dataset.

**Fig 2:**
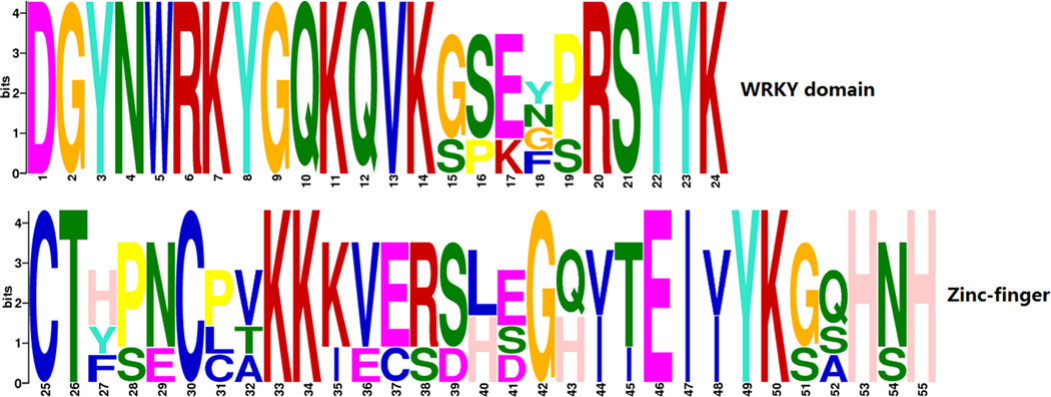
Domain prediction of thirteen WRKY protein sequences. The domain prediction, of thirteen *C*. *heterophylla* WRKY protein sequences and was performed using MEME software, which generated a letter logo to represent the WRKY domain and the zinc finger motif. The height of the letters in the y-axis represents the degree of conservation and relative frequency of each amino acid at that position.

**Fig 3:**
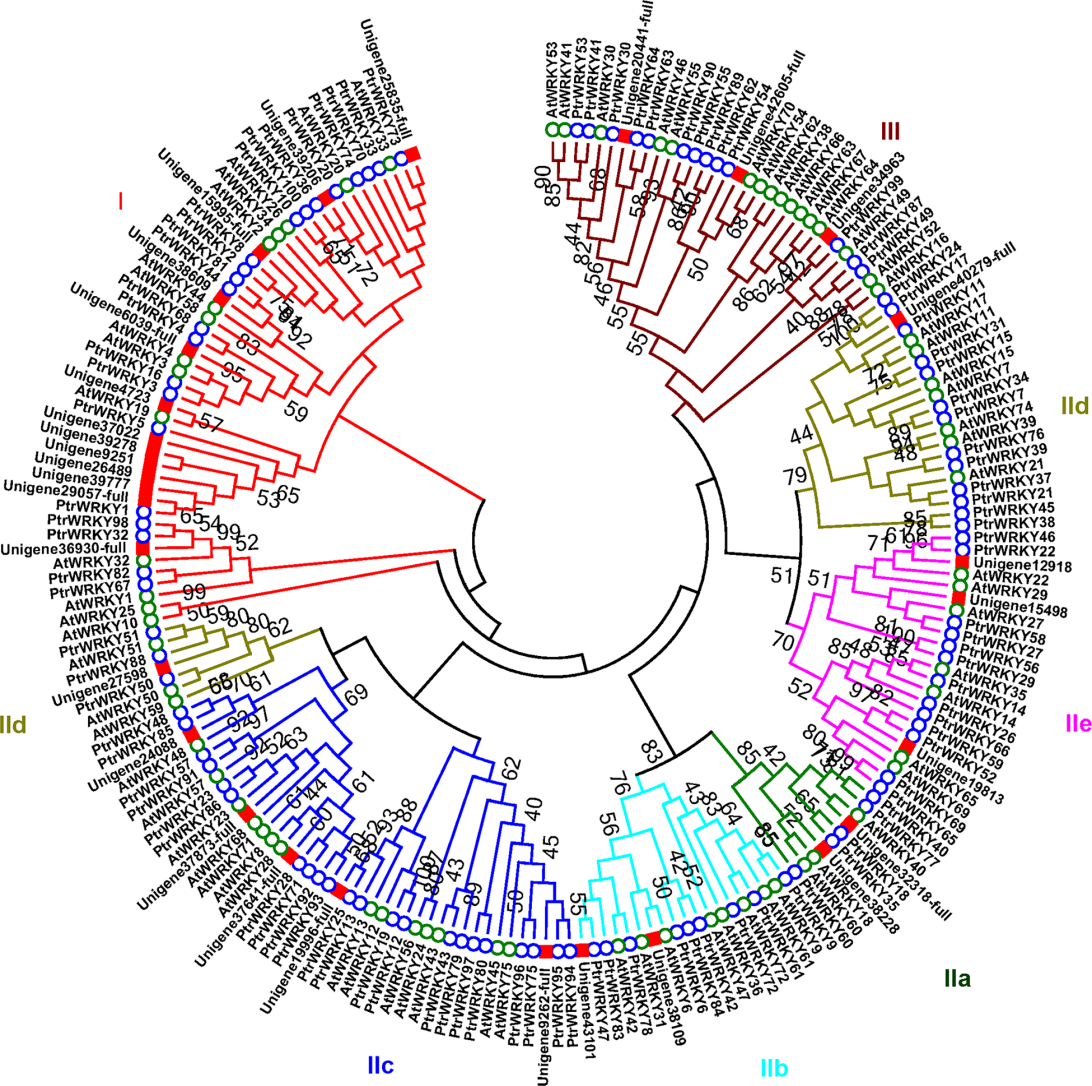
Phylogenetic analyses of WRKY proteins from *C*. *heterophylla*, *A*. *thaliana*, and *P*. *trichocarpa*. The phylogenetic tree of 30 putative *C*. *heterophylla* WRKYs, 72 AtWRKYs and 100 PtrWRKYs was constructed using MEGA 4.1, with a maximum likelihood (ML) method and 1,000 bootstrap replicates.

Following an analysis of the *C*. *heterophylla* floral bud transcriptome data set, we identified a total of 1,569 putative transcription factors and classified them into 64 families (Fig 1). Among them, 30 candidate genes encoding a WRKY domain were found and, using the Rapid Amplification of cDNA ends (RACE) technique, 13 full length genes with a complete open reading frame (ORF) were obtained (Table 1). Domain prediction clearly indicated that these proteins contained the conserved WRKY domain and zinc finger structure (Fig 2). To determine the phylogenetic relationships among the *C*. *heterophylla* WRKY proteins, an uprooted tree of all putative 30 ChWRKY proteins, 100 PtrWRKYs from *Populus trichocarpa*, and 72 AtWRKRs from *A*. *thaliana* was built using MEGA 4.1 (Fig 3). Based on the number of WRKY domains and the pattern of the zinc finger structures, the 30 corresponding WRKY proteins of *C*. *heterophylla* were divided into I, II and III groups. Group I contained 13 WRKY proteins, group III contained only 3 members with the specific zinc finger motif C_2_HC, while group II was further classified into five subgroups (IIa-e). Moreover, the phylogenetic tree was also constructed based on conserved WRKY domains. As shown in S1 Fig, group I contained sequences with a C-terminal WRKY domain or an N-terminal WRKY domain, and these sequences aligned within two different clusters, I-CT and I-NT, respectively. Of the thirty putative *WRKY* genes, fourteen belonged to group II, and one (*Unigene37641*) was chosen for further analysis. The full length cDNA of *Unigene37641* is 1,342 bp (S2 Fig), including a predicted 963 bp ORF, which encodes a 320 amino acids polypeptide with a relative molecular mass of 35.06 kDa. The Unigene37641 protein contains a conserved WRKY domain and a zinc finger motif (C-X_4_-C-X_23_-H-X_1_-H), and a BLASTX analysis of the *A*. *thaliana* WRKY proteins in NCBI database revealed that AtWRKY28 is the most closely related protein to Unigene37641. The characteristics of other candidate WRKYs are provided in S3 Table.

**Table 1:** Characteristics of *Corylus heterophylla* Fisch *WRKY* genes.

Unigene ID	Arabidopsis ortholog	*E*-value	cDNA length/ amino acids length	WRKY domain	Zinc finger motif	Subgroup
Unigene15995	AtWRKY2	0.00E+00	2241/746	WRKYGQK	C-X_4_-C-X_23_-H-X_1_-H	Group I
Unigene6039	AtWRKY3	1.00E-131	1170/389	WRKYGQK	C-X_4_-C-X_23_-H-X_1_-H	Group I
Unigene36930	AtWRKY32	8.00E-134	1515/504	WRKYGQK	C-X_4_-C-X_23_-H-X_1_-H	Group I
Unigene25835	AtWRKY33	4.00E-116	1797/598	WRKYGQK	C-X_4_-C-X_23_-H-X_1_-H	Group I
Unigene42605	AtWRKY49	3.00E-14	1005/334	WRKYGQK	C-X_4_-C-X_22_-H-X_1_-H	Group I
Unigene32318	AtWRKY40	3.00E-105	954/317	WRKYGQK	C-X_5_-C-X_23_-H-X_1_-H	Group II-a
Unigene19996	AtWRKY13	2.00E-63	717/238	WRKYGQK	C-X_4_-C-X_23_-H-X_1_-H	Group II-c
Unigene37873	AtWRKY23	9.00E-70	957/318	WRKYGQK	C-X_4_-C-X_23_-H-X_1_-H	Group II-c
Unigene37641	AtWRKY28	6.00E-77	963/320	WRKYGQK	C-X_4_-C-X_23_-H-X_1_-H	Group II-c
Unigene9262	AtWRKY75	1.00E-55	540/179	WRKYGKK	C-X_4_-C-X_23_-H-X_1_-H	Group II-c
Unigene29057	AtWRKY11	1.00E-134	1035/344	WRKYGQK	C-X_5_-C-X_23_-H-X_1_-H	Group II-d
Unigene40279	AtWRKY11	2.00E-100	816/271	WRKYGQK	C-X_5_-C-X_23_-H-X_1_-H	Group II-d
Unigene20441	AtWRKY53	2.00E-52	1086/361	WRKYGQK	C-X_7_-C-X_23_-H-X_1_-C	Group III

### Expression analyses of *WRKY* genes during the overwintering

**Fig 4:**
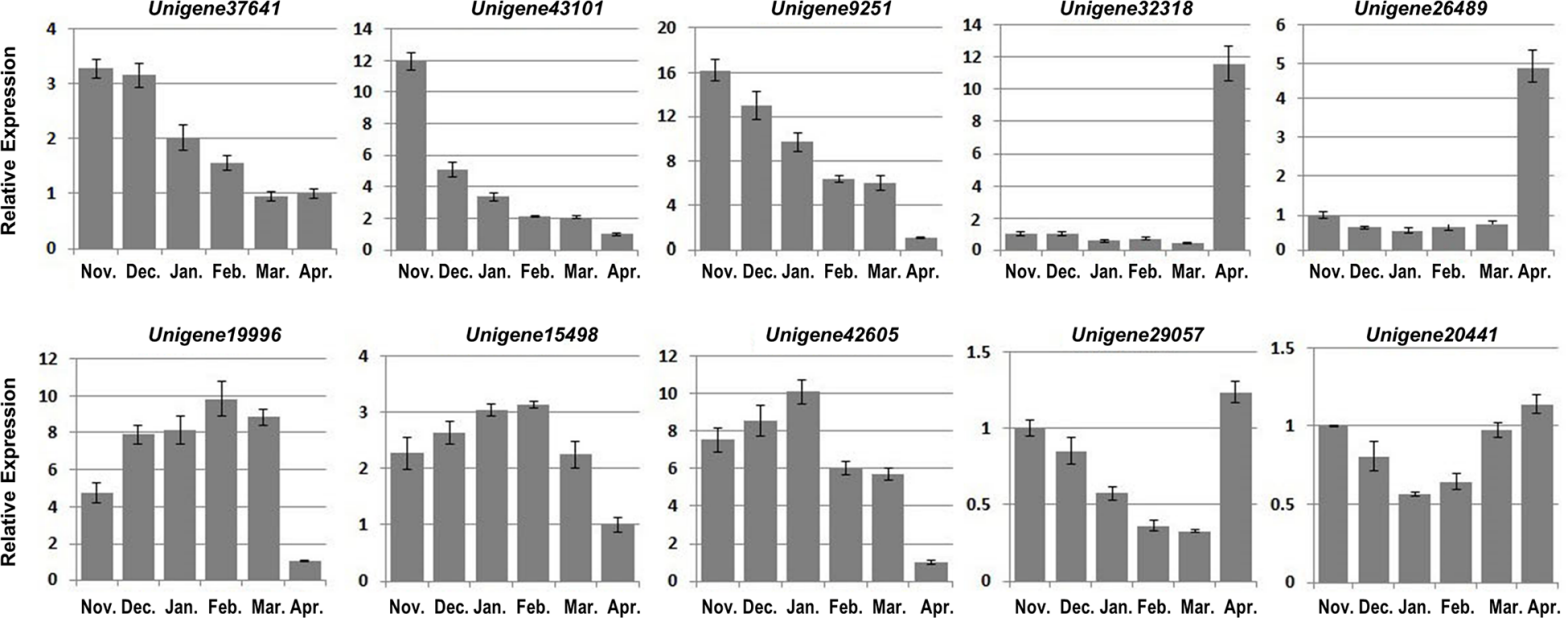
qRT-PCR analysis of *WRKY* gene expression during the overwintering. *C*. *heterophylla* Fisch floral buds were collected each month from 2011 November to 2012 April. The *ChActin* gene was used as an internal control for qRT-PCR analysis. The relative expression (y-axis) was calculated using the 2^−ΔΔCt^ formula. The mean value and standard error were obtained from three biological and three technical replicates.

We evaluated the expression patterns of the 30 candidate *WRKY* genes in floral buds from *C*. *heterophylla* plants grown under normal conditions during the overwintering period, from November to April, by quantitative PCR (qRT-PCR) analysis. The *WRKY* genes were divided into four types according to their expression patterns (Fig 4 and S3 Fig): Type I (*Unigene37641*, *Unigene43101*, *Unigene9251*, *Unigene4723*, *Unigene15995*, *Unigene40279*, *Unigene12918*, *Unigene37873*, *Unigene34963*, *Unigene38109*, *Unigene36930* and *Unigene9262*) showed the highest expression in November, which then declined until April; Type II (*Unigene19996*, *Unigene15498*, *Unigene42605*, *Unigene37022*, *Unigene39206*, *Unigene39278*, *Unigene39777*, *Unigene25835*, *Unigene19813*, *Unigene38228*, *Unigene38609* and *Unigene24088*) showed an initial increase in expression followed by a subsequent decrease; Type III (*Unigene32318*, *Unigene26489* and *Unigene27598*) low expression levels initially, followed by a significant increase in April; and Type IV (*Unigene29057*, *Unigene20441* and *Unigene6039*) showed a decline after a general trend of increasing expression.

### Expression patterns of *ChWRKYs* under cold, drought and salinity stresses

**Fig 5:**
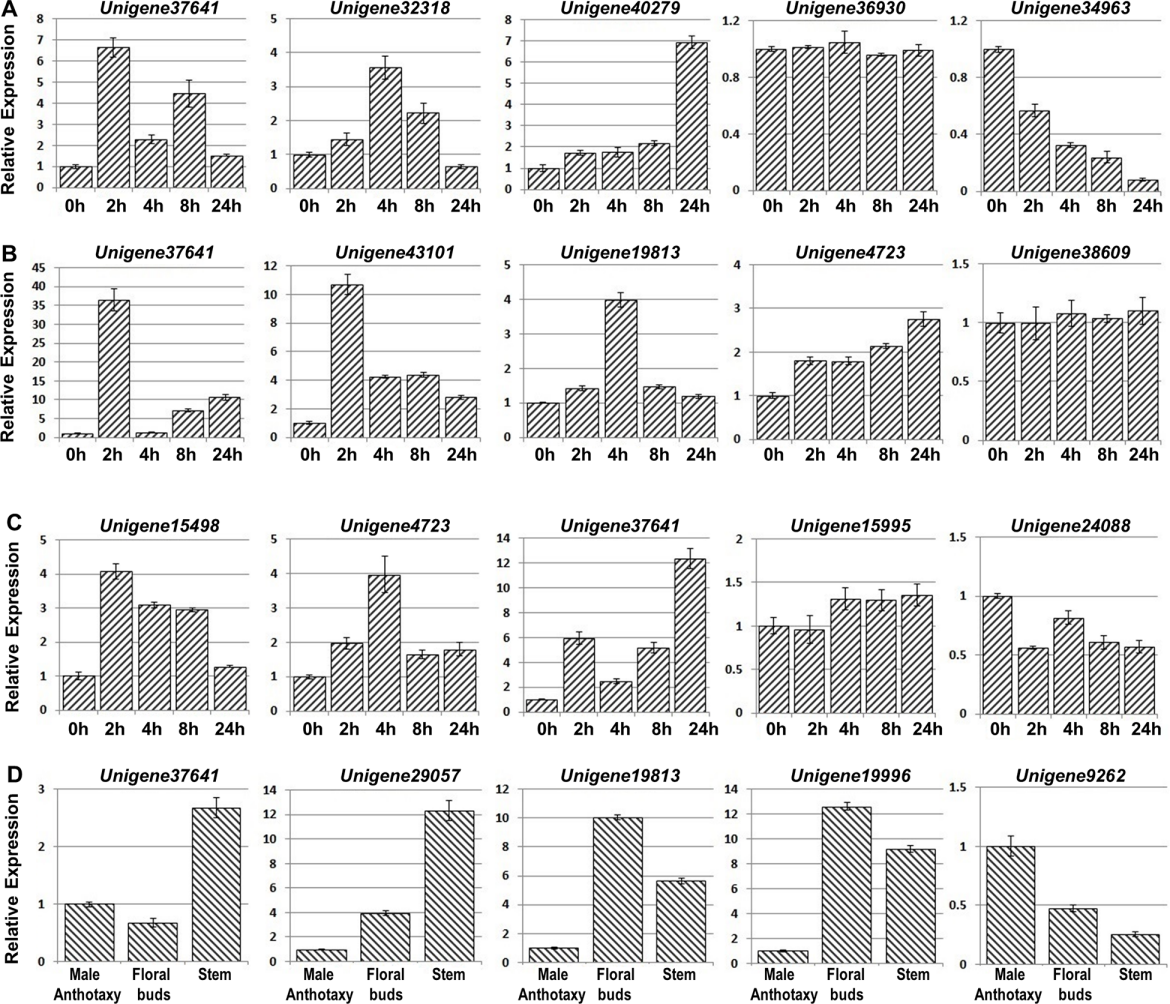
Expression patterns of *WRKY* genes after exposure to various abiotic stresses and in different organs/tissues. The *ChActin* gene was used as an internal control for qRT-PCR. The y-axis represents relative expression, calculated using the 2^−ΔΔCt^ formula. (**A-C**) Expression profiles of *WRKY* genes under cold (4°C), drought (25% PEG6000) and salinity (400mM NaCl) growth conditions, respectively. Leaf samples were collected at 2, 4, 8 and 24h, with the untreated seedlings grown and analyzed in parallel. The expression level values of the genes in untreated seedlings were set to 1.0. (**D**) Expression patterns of *WRKY* genes in different organs/tissues, including male anthotaxies, floral buds and stems, which were collected at the same stage. The experiments were repeated with at least three biological and three technical replicates, yielding consistent results.

To identify the potential functions of the 30 *WRKY* genes in response to external stimuli, their expression profiles were analyzed in *C*. *heterophylla* leaves following cold, drought and salinity treatments. At 4°C, the expression of *Unigene37641*, *Unigene32318*, *Unigene9262* and *Unigene39278* was up-regulated with a maximal increase in expression of 6.7, 3.6, 3.6 and 7.0 fold after 2, 4, 8 and 24h, respectively (Fig 5A and S4 Fig). Similarly, 28 of the *WRKY* genes were up-regulated and reached maximum expression levels from 2-24h after the onset of a treatment with NaCl (Fig 5C and S6 Fig), while a drought treatment, induced by the application of polyethylene glycol (PEG6000), resulted in the up-regulation of 21 *WRKY* genes, which peaked at 2h (Fig 5B and S5 Fig). The expression of *Unigene36930*, *Unigene38609* and *Unigene15995* did not change as a consequence of the cold, drought or salinity treatments (Fig 5), but the expression of *Unigene34963* and *Unigene24088* was down regulated by the cold and salinity treatment, respectively (Fig 5A and 5C). Of the 30 *ChWRKY* genes, 23 were up-regulated by all three abiotic stresses (S4 Table), including *Unigene37641*, which was selected for further functional analysis by over-expression in transgenic *A*. *thaliana* plants.

### Expression patterns of *WRKY* genes in different organs/tissues under cold growth conditions

The expression of all 30 *WRKY* genes was evaluated in the male anthotaxy, floral buds and stems (Fig 5D and S7 Fig). The expression levels of *Unigene37641* and *Unigene29057* in stems, and of *Unigene19813* and *Unigene19996* in floral buds, were 2.7, 12.4, 10.1 and 12.6 fold greater, respectively, than in the male anthotaxy (Fig 5D), while the expression of *Unigene9262* in the male anthotaxy was 3.9 and 2.1 fold greater, respectively, than in stems and floral buds. The expression analysis also revealed considerable variation in WRKY gene expression among the three organs/tissues, with 18 genes being highly expressed in stems and 10 in floral buds. In contrast, only 2 *WRKY* genes (*Unigene9262* and *Unigene43101*) were abundantly expressed in the male anthotaxy.

### Unigene25835, Unigene37641 and Unigene20441 localized in the nucleus

**Fig 6:**
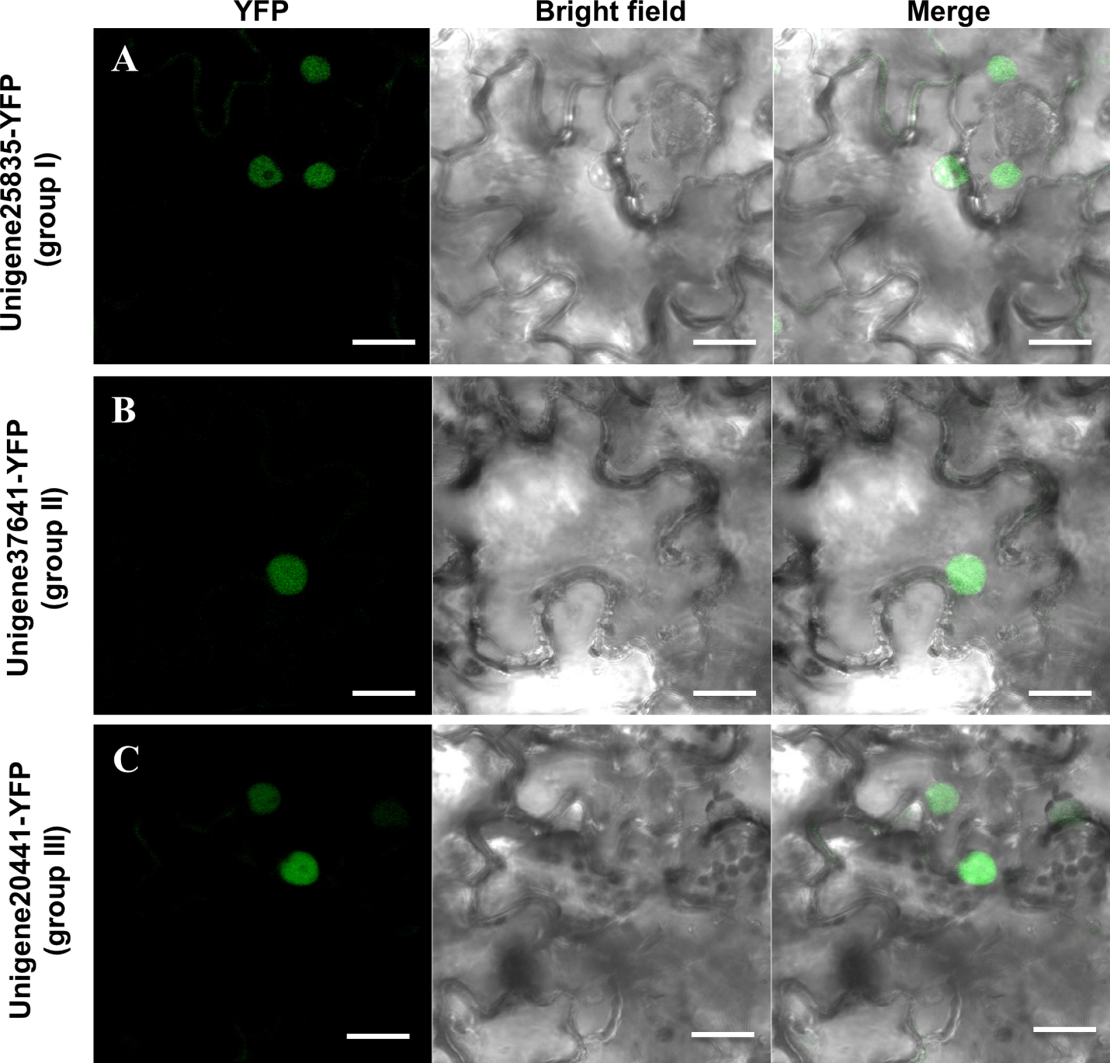
Subcellular localization of WRKY proteins. Confocal images of *Nicotiana benthamiana* epidermal leaf cells expressing Group I Unigene25835 (A), Group II Unigene37641 (B) and Group III Unigene20441 (C) WRKY proteins fused to yellow fluorescent protein (YFP). Scale bar = 20 μm.

Computational analysis using the Euk-mPLoc software predicted that Unigene25835 (group I), Unigene37641 (group II) and Unigene20441 (group III) localize to the nucleus (S5 Table). To confirm their subcellular localization, we transiently expressed the proteins, each fused to the yellow fluorescent protein (YFP) reporter, in *Nicotiana benthamiana* leaf abaxial epidermal cells, under the control of the constitutive 35S promoter. As predicted, each of the fusion proteins was observed to exclusively accumulate in the nucleus of the epidermal cells (Fig 6), suggesting that Unigene25835, Unigene37641 and Unigene20441 are nuclear proteins, in accordance with their predicted function as transcription factors.

### Overexpression of *Unigene37641* enhanced cold tolerance in *A*. *thaliana*

**Fig 7:**
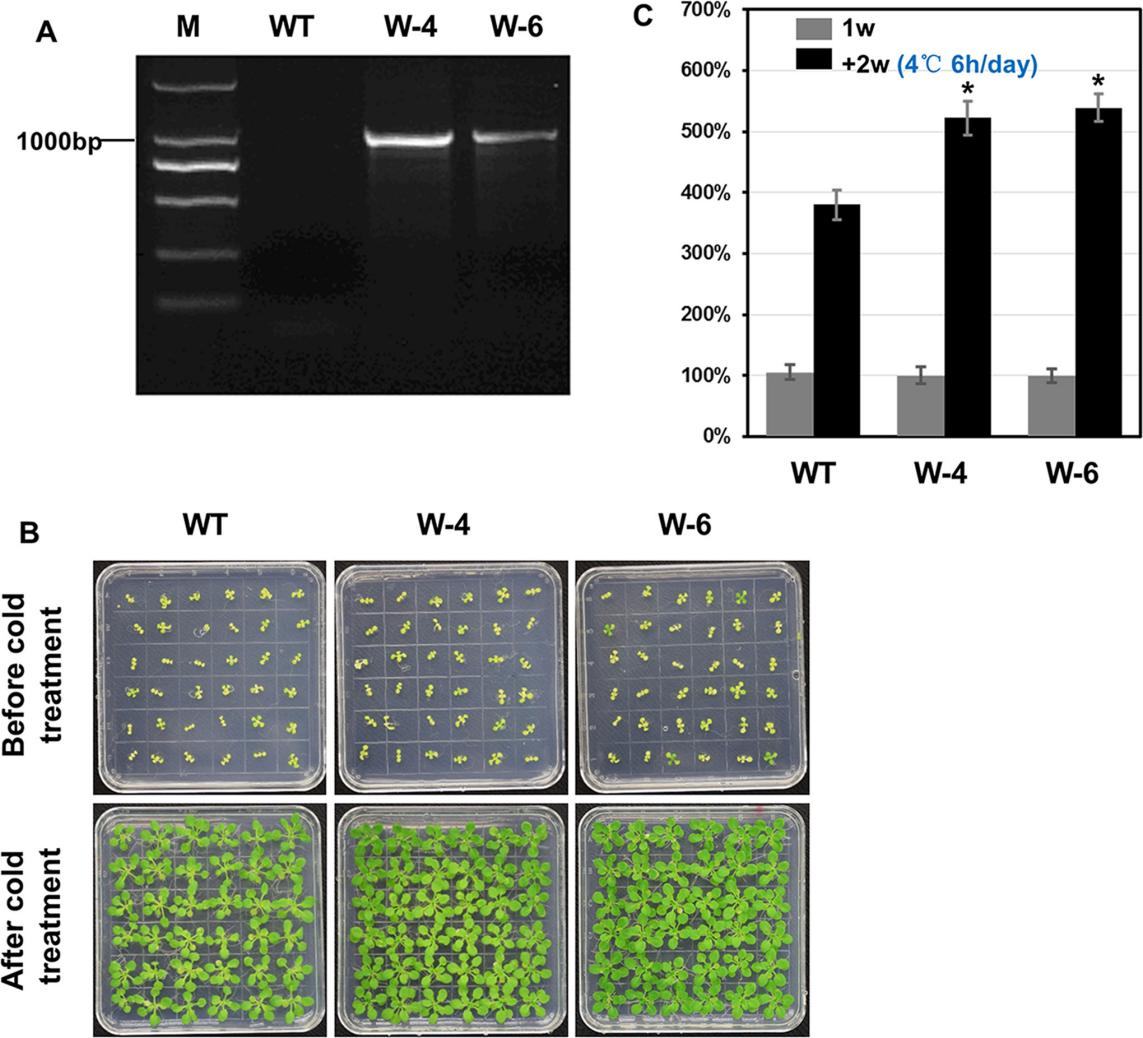
*Unigene37641* overexpression enhanced cold tolerance in transgenic *A*. *thaliana*. (A) Expression level of *Unigene37641* in T_2_ *Arabidopsis* transgenic lines (W-4 and W-6) and wild type (WT) plants using RT-PCR. (B) The WT and T_2_ plants were cultured on MS medium for 1 week were shown in top row. Seedlings were grown at 4°C (6 h per day) for 2 weeks were shown in bottom row. (C) Comparison of seedling fresh weight after cold treatment. The mean value of fresh weights and the standard errors were calculated based on three replicates.

To investigate the function of *Unigene37641*, we overexpressed the gene in *A*. *thaliana*. RT-PCR analysis showed that two T_2_ transgenic lines (W-4 and W-6) had high levels of expression of the *Unigene37641* gene, while expression was not detected in wild type plants (Fig 7A). After cold treatment for 2 weeks, seedlings of the two *Unigene37641* overexpressing transgenic lines showed more vigorous growth than the wild type (WT) seedlings and this was confirmed by a quantitative analysis of the average fresh weight (Fig 7B and 7C). A cold tolerance test further showed higher levels of soluble protein and greater SOD and POD activities in the transgenic lines than in WT plants (S8A–S8C Fig). Moreover, MDA levels, which provide a measure of the degree of damage to cell membranes caused by lipid peroxidation, were substantially lower in the transgenic lines that in WT plants following cold stress (S8D Fig). These results suggest that overexpression of *Unigene37641* enhanced cold stress tolerance in the transgenic *A*. *thaliana*.

## Discussion

Plant WRKY transcription factors comprise a superfamily involved in the regulation of a variety of development processes and stress responses [57, 68]. However, compared with the considerable progress that has been made in understanding their role in biotic stresses, less is known about their function in the context of abiotic stresses [29]. Numerous WRKY transcription factors have been identified in various plant species, such as *A*. *thaliana*, *Oryza sativa* (rice), *P*. *trichocarpa* (poplar), *Glycine max* (soybean) and *Pinus monticola* (pine) [69–72] and there is considerable interest in investigating their role in responses to factors such as drought, high salinity and cold temperature, in the context of effects on crop yield and quality [73]. In this study, we investigated the composition and potential functions of the WRKY gene family of the hazelnut tree *C*. *heterophylla* Fisch, an economically and ecologically species with several traits that make it valuable as an agricultural commodity, one of which being its hardiness in cold temperatures. A total of 30 putative *WRKY* unigenes were identified, of which 13 members were cloned to obtain full length sequences with complete ORFs, and they were all found to have conserved structural features, including WRKY domains and zinc finger regions (Fig 2).

A phylogenetic tree of WRKY proteins from *C*. *heterophylla*, *A*. *thaliana*, and *P*. *trichocarpa* was constructed to examine their evolutionary relationships (Fig 3). According to the report by Eulgem *et al* [32], all these corresponding proteins could be classified into three main groups (I, II and III) based on the number of WRKY domains and the type of zinc-finger motif (Table 1). Interestingly, the phylogenetic analyses demonstrated that Unigene42605 sequence aligned within clade III. However, this protein has a C_2_H_2_ zinc finger structure that is different from the typical C_2_HC sequence that is typical of type of group III (Fig 3 and S9 Fig). Based on the phylogenetic tree of conserved WRKY domains and alignment analyses, Unigene42605, which contained a C-X_4_-C-X_22_-H-X_1_-H motif, should be assigned to group I (S1 and S9 Figs). Furthermore, Unigene29057 clustered in subgroup IId based on the conserved WRKY domains (S1 Fig). This finding was inconsistent with the results shown in Fig 3, where this protein was classified in group I. The sequence alignments demonstrated that Unigene29057 contained only one WRKY domain with a C-X_5_-C-X_23_-H-X_1_-H zinc finger, implying that this member should belong to group IId (S9 Fig). A phylogenetic tree combining WRKYs from different species has the potential to not only elucidate the evolutionary relationships of the proteins [70], but also to allow predictions of the functions of the *C*. *heterophylla* proteins based on the functional clades of the orthologous proteins, since close homologs can share expression profiles and functions [74]. For example, AtWRKY28 and PtrWRKY28 aligned within group IIc (Fig 3), members of which are associated with responses to abiotic stresses [70, 75]. Thus, we can infer that Unigene37641 might have similar roles since it clustered with the same group. Subcellular localization studies showed that Unigene25835-YFP (group I), Unigene37641-YFP (group II) and Unigene20441-YFP (group III) fusion proteins accumulated in the nucleus of *N*. *benthamiana* leaf epidermal cells following transient expression (Fig 6), which is in accordance with their computationally predicted localization (S5 Table) and previous studies of other species [76].

We next examined the expression patterns of the WRKY transcription factors in the floral buds of overwintering *C*. *heterophylla*. It has previously been determined that the floral buds undergo four developmental stages during winter: NA (non-cold acclimation), CA (cold acclimation), MW (midwinter) and DA (de-acclimation) [25]. The expression data showed that several *WRKY* genes, such as *Unigene37641*, *Unigene43101* and *Unigene9251*, were highly expressed in the early CA stage in November, with a subsequent decrease in expression (Fig 4). These *WRKY* genes may be responsible for activating the expression of downstream target genes to enhance cold tolerance of the floral buds during the winter season. *Unigene19996*, *Unigene15498* and *Unigene42605* showed a different expression pattern, in that they showed the highest transcript levels at the MW stage, suggesting an involvement in later cold acclimation. In contrast, *Unigene32318*, *Unigene26489* and *Unigene27598* were not upregulated from November to March but showed a substantial increase in expression in April (Fig 4 and S3 Fig), suggesting functions other than responses to low temperatures [25]. These genes may be induced by other environmental cues, such as short photoperiods, and contribute to developmental processes [27] and/or flowering time regulation [77]. Based on the expression profiles, we hypothesize that *Unigene32318*, *Unigene26489* and *Unigene27598* may be involved in floral bud development, since *C*. *heterophylla* floral buds germinate in April. Finally, the expression of *Unigene29057* and *Unigene20441* showed an initial decline followed by an increasing trend (Fig 4); however, since individual *WRKY* genes have been found to function in both environmental stimuli and plant development [27, 73], it may be that these two genes are involved in both cold acclimation and flower development.

To assess the potential influence of other environmental factors on the observed expression patterns of the *WRKY* genes, we induced other abiotic stresses on the plants using an artificial climate chamber. qRT-PCT analyses revealed that 24 *WRKY* genes were significantly up-regulated and 2 down-regulated by cold stress (S4 Table). Interestingly, the expression of *Unigene32318* and *Unigene26489* was substantially induced by the cold treatment, peaking after 4h (Fig 5A and S4 Fig), even though their expression was not observed to increase from November to March under overwintering conditions (Fig 4). This suggests that their expression is induced by multiple factors, potentially involving several signaling pathways, implying a complex regulatory network [29]. Of the 30 *WRKY* genes identified here, the expression of 28 changed following the drought and salt treatments (S4 Table), indicating a general correlation between drought and salinity stress responses, and the existence of crosstalk between the respective signal transduction pathways [71, 78]. As shown in Fig 5, some of the *WRKY* genes showed an extremely rapid response to abiotic stress: the expression of *Unigene37641*, *Unigene43101* and *Unigene15498* peaked at 2h after the cold, drought and salinity treatments, respectively. We note that an increasing number of reports describe rapid expression of WRKY transcription factors in association with stress tolerance [32, 73]. When the expression profiles of the 30 *WRKY* genes were evaluated in three different organs/tissues, 18 were most highly expressed in the stem and only 2 genes were strongly expressed in the male anthotaxy. This difference in organ/tissue specific expression suggests functional divergence of the respective genes [30].

We observed that the expression of *Unigene37641* was significantly up-regulated by all three stresses and we selected this gene for functional analysis by overexpression in *A*. *thaliana*. Several previous studies have reported that overexpression of *WRKY* genes in *A*. *thaliana* can enhance its tolerance to various abiotic stresses [36], one example being the overexpression of soybean *GmWRKY21*, which led to enhanced cold tolerance [71]. Another example came from the overexpression of the rice *OsWRKY45* and *OsWRK72* genes, which conferred drought and salt tolerance to transgenic *A*. *thaliana* plants [79, 80]. Compared with WT plants, transgenic *A*. *thaliana* overexpressing *Unigene37641* showed no obvious phenotypic differences. However, the seedlings were less susceptible to cold stress (Fig 7B), which is in accordance with a previous report in which grapevine *VpWRKY2* was overexpressed in *A*. *thaliana*, resulting in enhanced cold stress tolerance [57]. *Unigene37641* overexpressing plants also showed levels of higher soluble protein, SOD and POD activities, and lower MDA levles compared to WT controls following the cold treantment (S8 Fig). This suggests that *Unigene37641* may help protect the plant by a mechanism that includes reducing membrane damage that would result in increased MDA production [81]. Several studies have shown similar expression patterns of putative *WRKY* genes and orthologs from *A*. *thaliana* [74], and overexpression of *AtWRKY28* in *A*. *thaliana* was recently shown to enhance tolerance to various stresses, including drought, salinity, oxalic acid and fungal pathogens, suggesting diverse regulatory functions [75, 82, 83]. Given that, of the 30 *C*. *heterophylla WRKY* genes, *Unigene37641* is the most closely related to *AtWRKY28*, we propose that *Unigene37641* may exhibit similar expression patterns and possibly have similar functions [74].

In conclusion, the regulatory mechanisms of WRKY proteins involved in stress tolerance are complex and further studies are needed to elucidate their functions. This current investigation of *WRKY* genes expression in *C*. *heterophylla* also provides a platform for further exploring the function of *WRKY* genes in other species and suggests candidate genes for enhancing biotic and abiotic stress tolerance in crops.

## Supporting Information

S1 Fig Phylogenetic analyses of *C*. *heterophylla* WRKY domains. A phylogenetic tree of conserved WRKY domains, built using MEGA 4.1 and employing the neighbour-joining (NJ) method with 1,000 bootstrap replicates. Group I was clustered into two groups, I-CT and I-NT, based on the C-terminal WRKY domain and N-terminal WRKY domains, respectively.
(TIF)

S2 Fig Sequence analysis of the *Unigene37641* cDNA. The cysteine and the histidine residues of the zinc-finger motif are boxed and the shaded area represents the WRKY domain.
(TIF)

S3 Fig Expression profiles of *WRKY* genes in *C*. *heterophylla* Fisch grown under normal conditions. (TIF)

S4 Fig Expression profiles of *WRKY* genes in *C*. *heterophylla* Fisch under cold stress treatment. (TIF)

S5 Fig Expression profiles of *WRKY* genes in *C*. *heterophylla* Fisch under drought stress treatment. (TIF)

S6 Fig Expression profiles of *WRKY* genes in *C*. *heterophylla* Fisch under salinity stress treatment. (TIF)

S7 Fig Expression profiles of *WRKY* genes in different organs/tissues of *C*. *heterophylla* Fisch. (TIF)

S8 Fig Analysis of enhanced cold tolerance in transgenic *A*. *thaliana* lines overexpressing *Unigene37641*. Six-week-old wild type (WT) and T_2_ transgenic lines (W-4, W-6) were held at 4°C for 24 h. (A) Soluble protein content in WT and T_2_ transgenic leaves exposed to cold. (B) Superoxide dismutase activity. (C) Peroxidase activity. (D) Malondialdehyde content. The mean values and standard errors were derived from three experimental replicates.
(TIF)

S9 Fig Sequence alignment analyses of WRKY domains. Based on the features of their WRKY domains, the corresponding proteins from *C*. *heterophylla* and *P*. *trichocarpa* were divided into three groups. Groups II was further classified into five subgroups (IIa, IIb, IIc, IId, IIe). The WRKYGQK domains are indicated with red boxes and the zinc-finger motif sequences are indicated with red triangles.
(TIF)

S1 Table Primers used for RACE-PCR analysis. (DOCX)

S2 Table Primers used for *C*. *heterophylla* Fisch *WRKYs* expression pattern analysis. (DOCX)

S3 Table Characteristics of *WRKY* genes in *C*. *heterophylla* Fisch. (DOCX)

S4 Table *WRKY* expression patterns under abiotic stress. (DOCX)

S5 Table Subcellular localization prediction using Euk-mPLoc. (DOCX)
