# Supplementary figures and images for: Expression and Functional Analysis of WRKY Transcription Factors in Chinese Wild Hazel, Corylus heterophylla Fisch
Source: PLoS One. 2015 Aug 13;10(8):e0135315. doi: 10.1371/journal.pone.0135315 (PMC4536078; doi:10.1371/journal.pone.0135315)

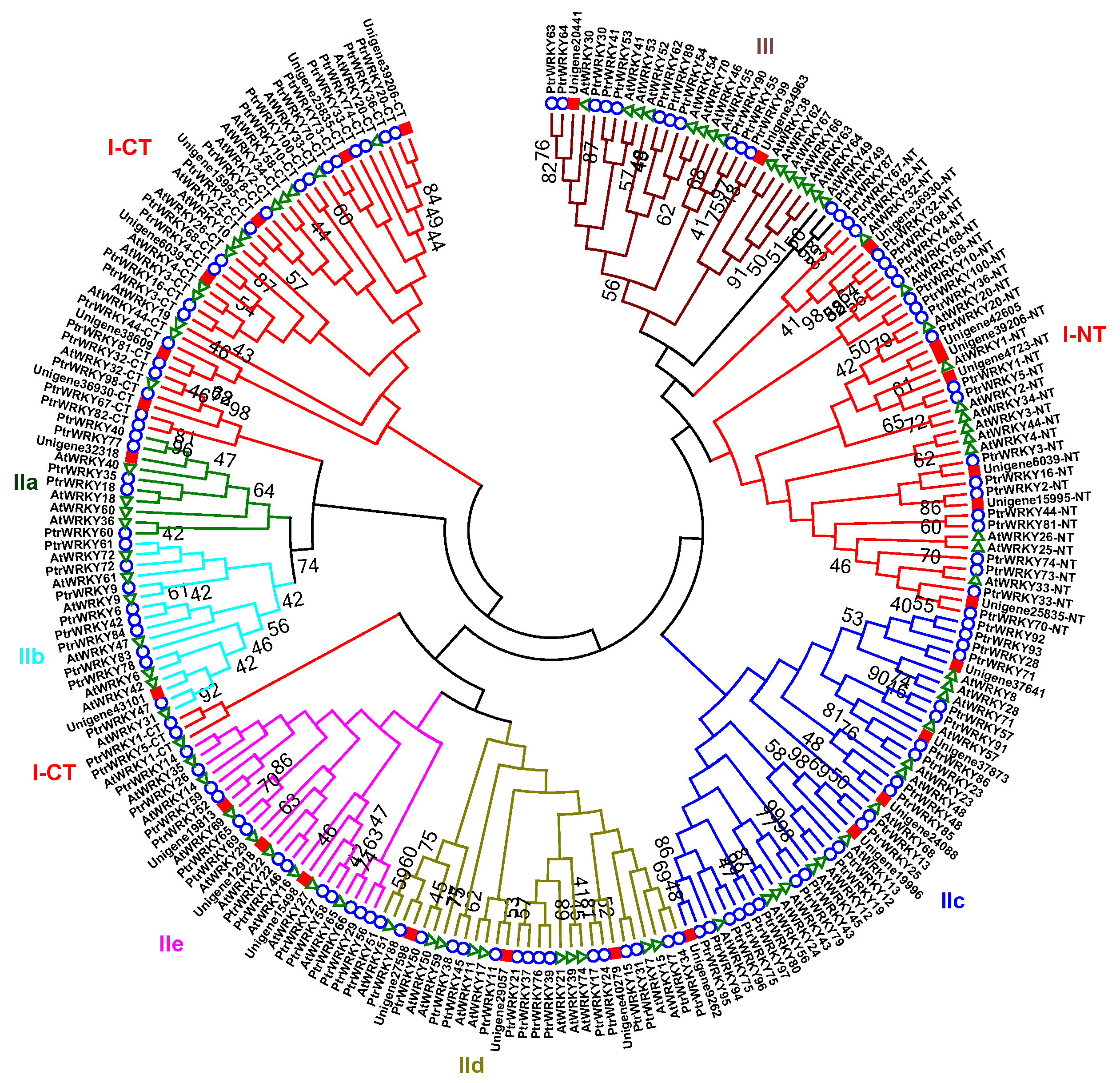

Supplement: S1 Fig — A phylogenetic tree of conserved WRKY domains, built using MEGA 4.1 and employing the neighbour-joining (NJ) method with 1,000 bootstrap replicates. Group I was clustered into two groups, I-CT and I-NT, based on the C-terminal WRKY domain and N-terminal WRKY domains, respectively. (TIF) [file pone.0135315.s001.tif]

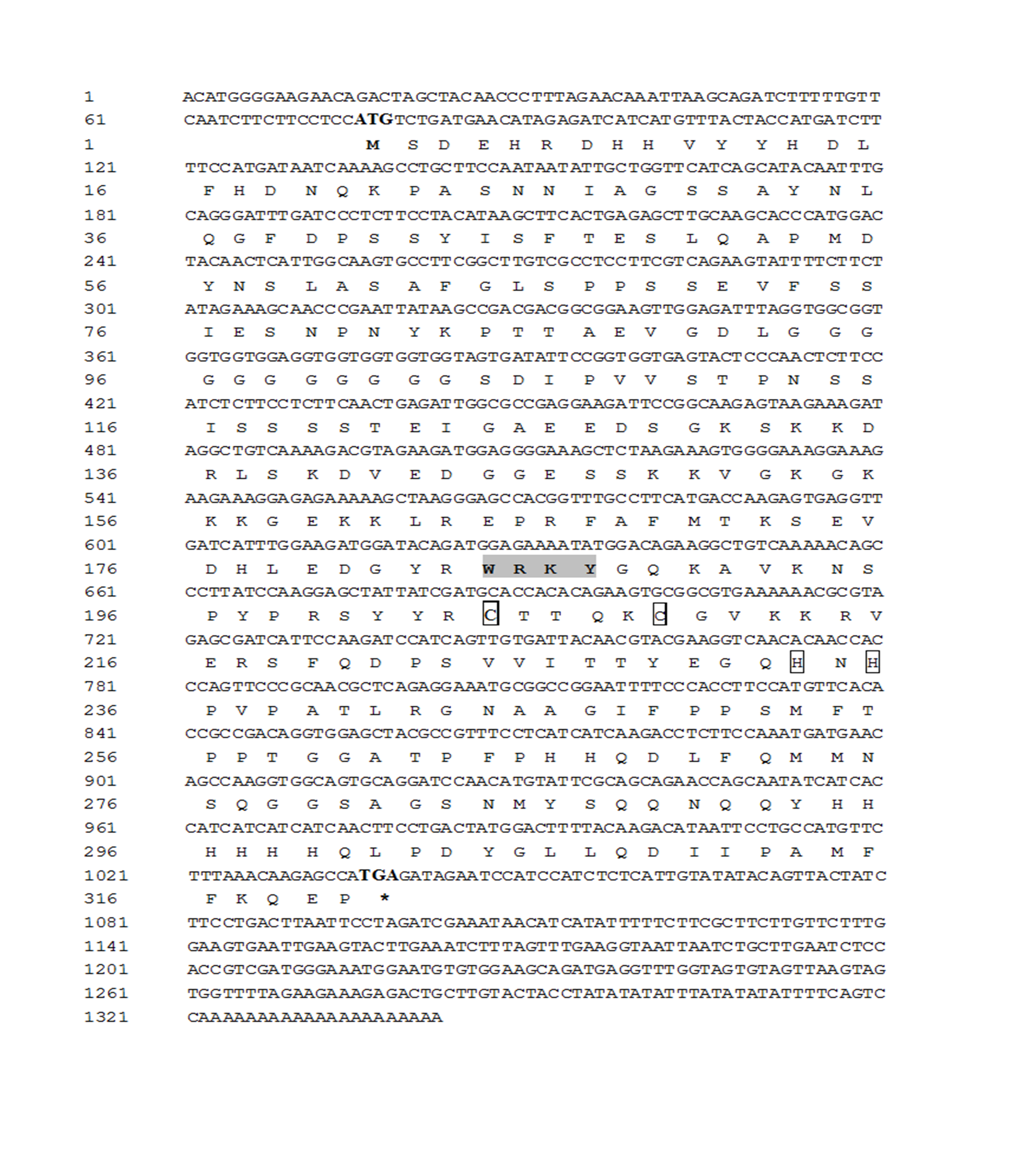

Supplement: S2 Fig — The cysteine and the histidine residues of the zinc-finger motif are boxed and the shaded area represents the WRKY domain. (TIF) [file pone.0135315.s002.tif]

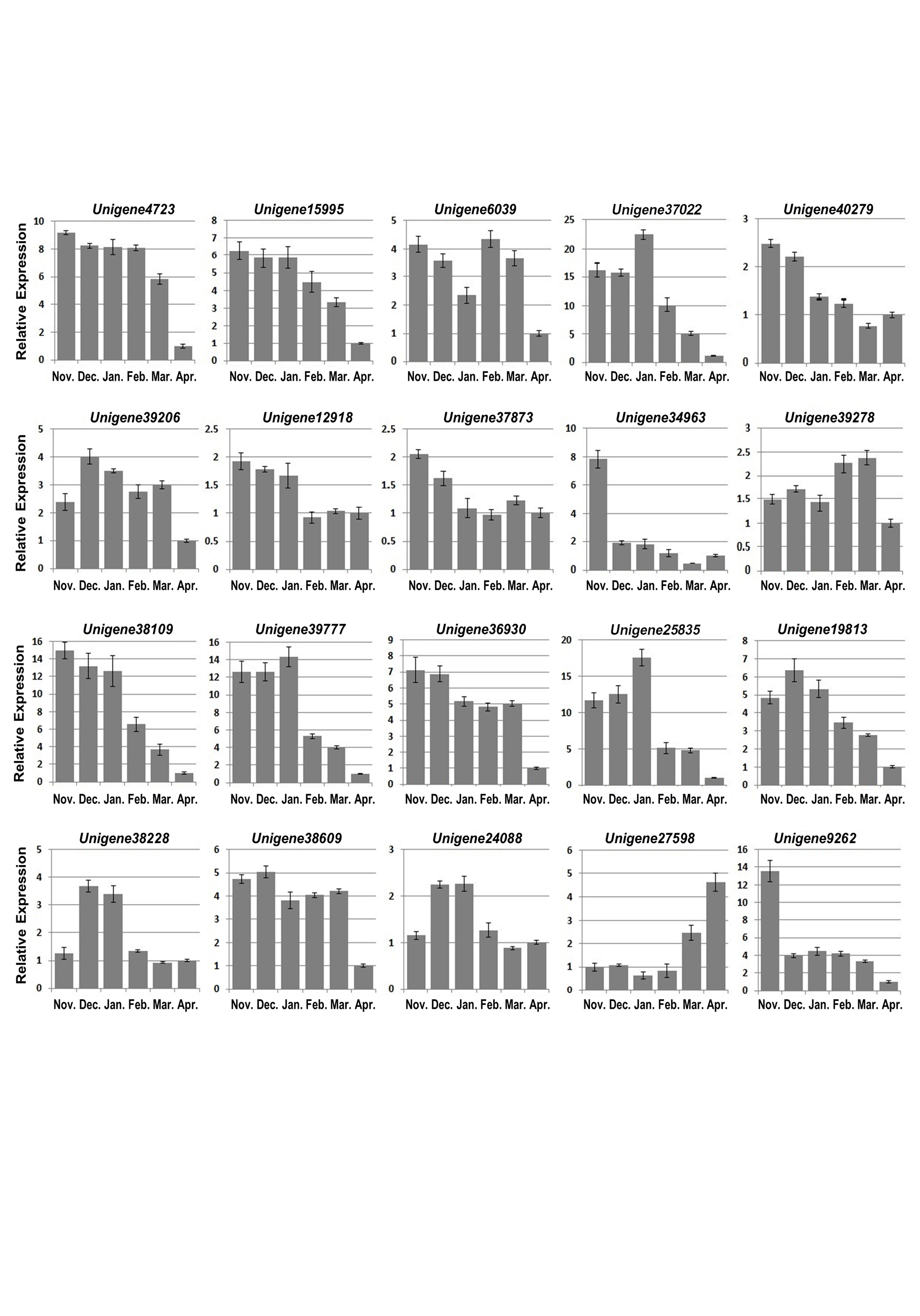

Supplement: S3 Fig — (TIF) [file pone.0135315.s003.tif]

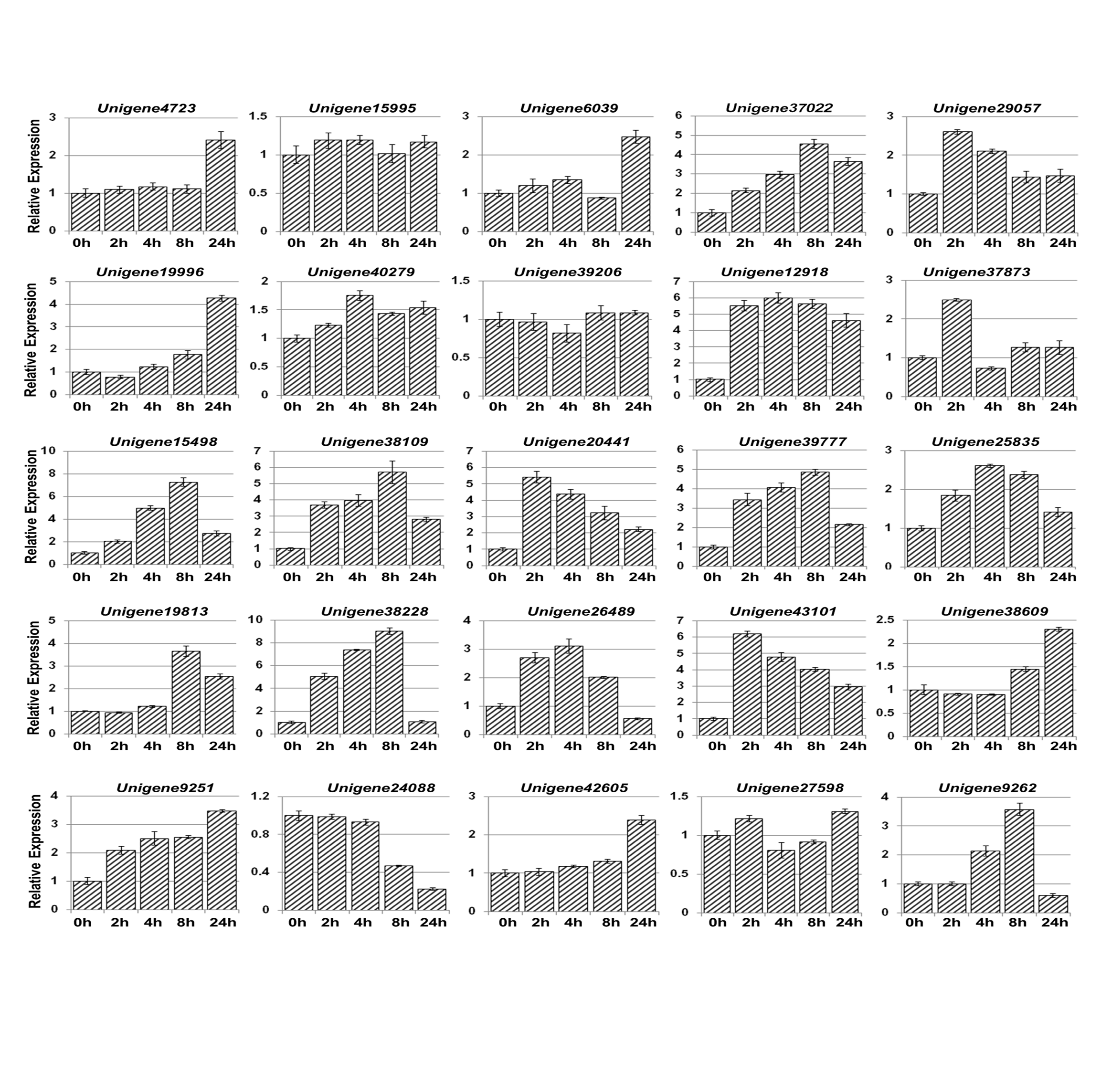

Supplement: S4 Fig — (TIF) [file pone.0135315.s004.tif]

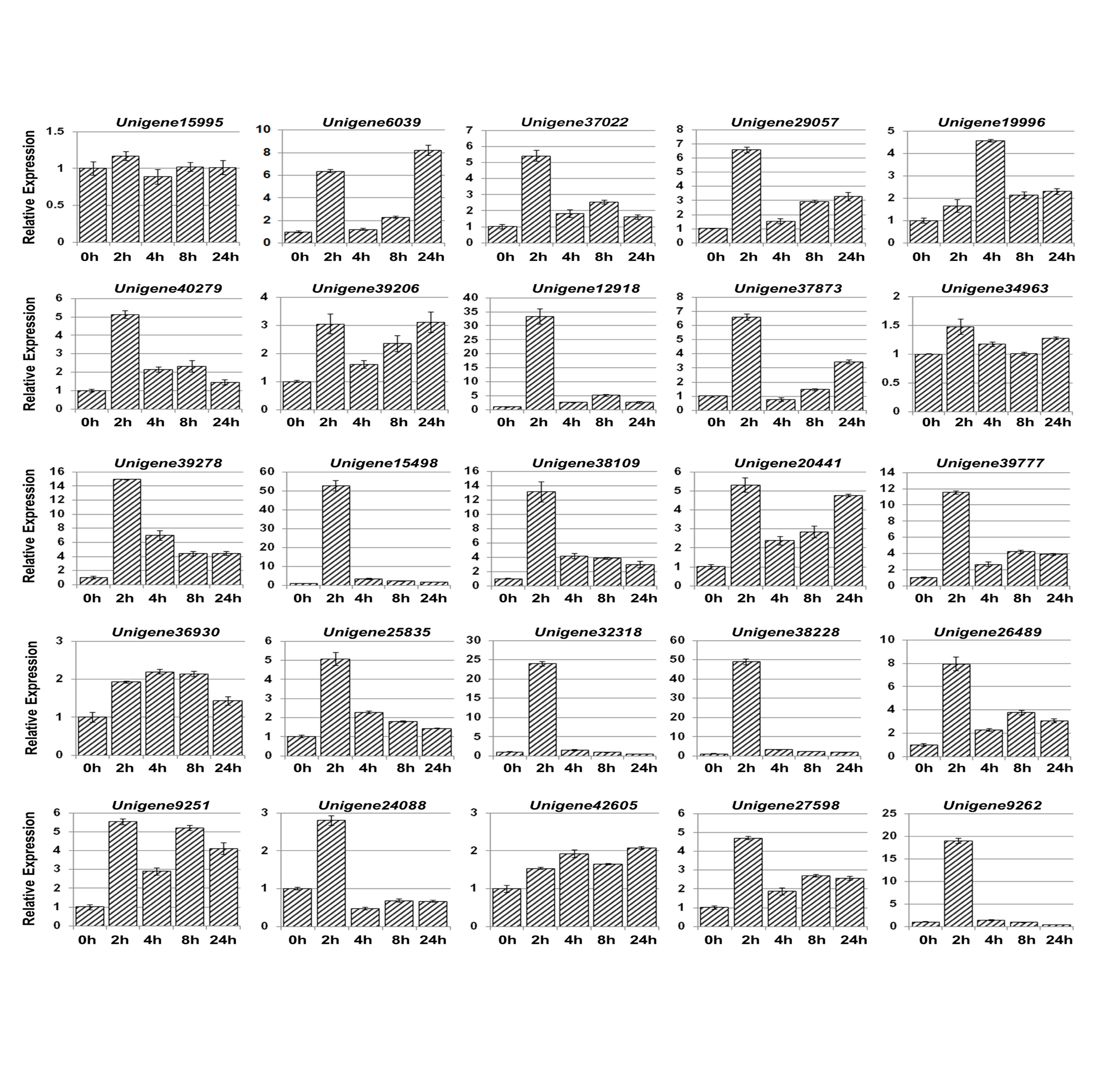

Supplement: S5 Fig — (TIF) [file pone.0135315.s005.tif]

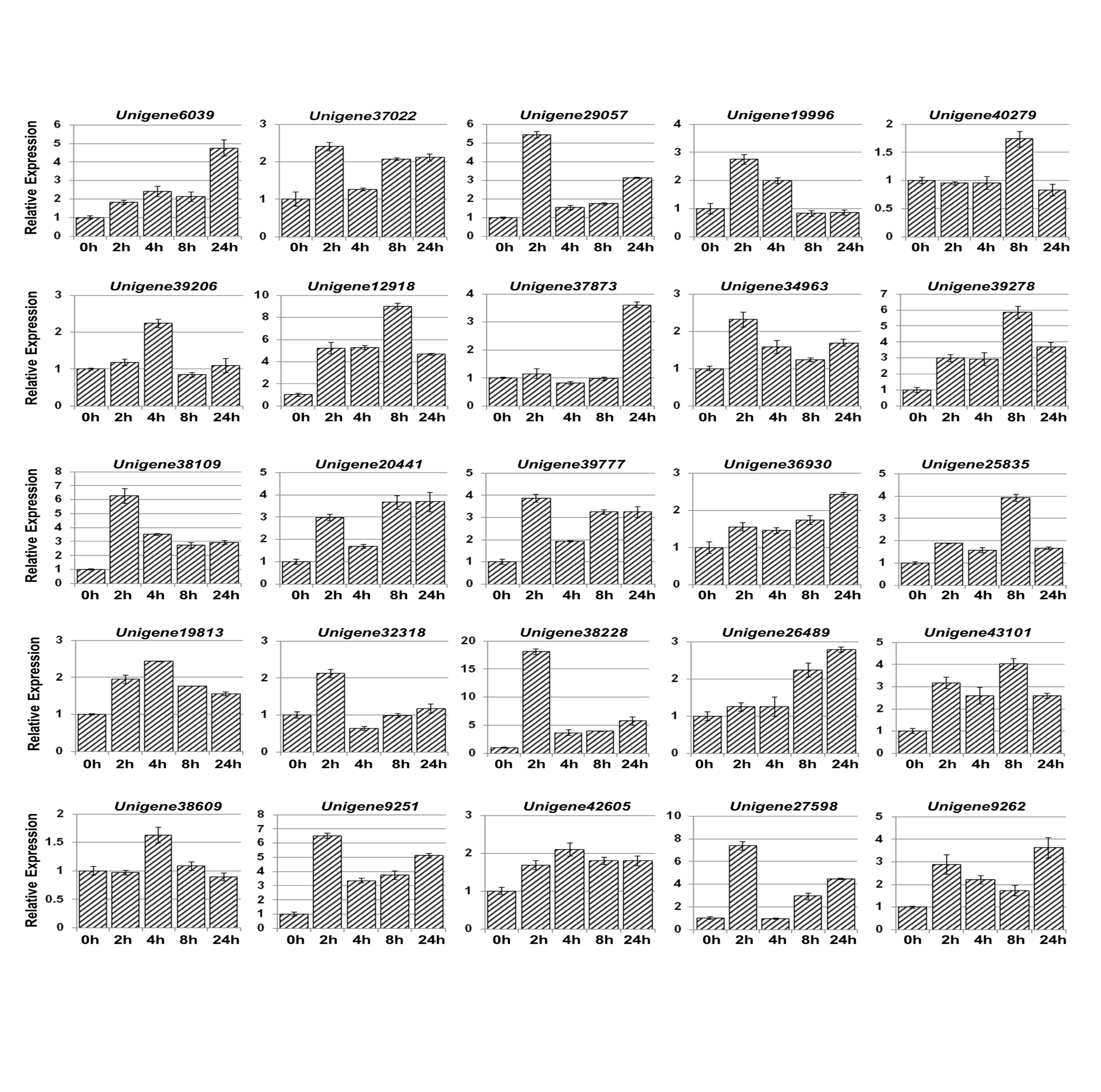

Supplement: S6 Fig — (TIF) [file pone.0135315.s006.tif]

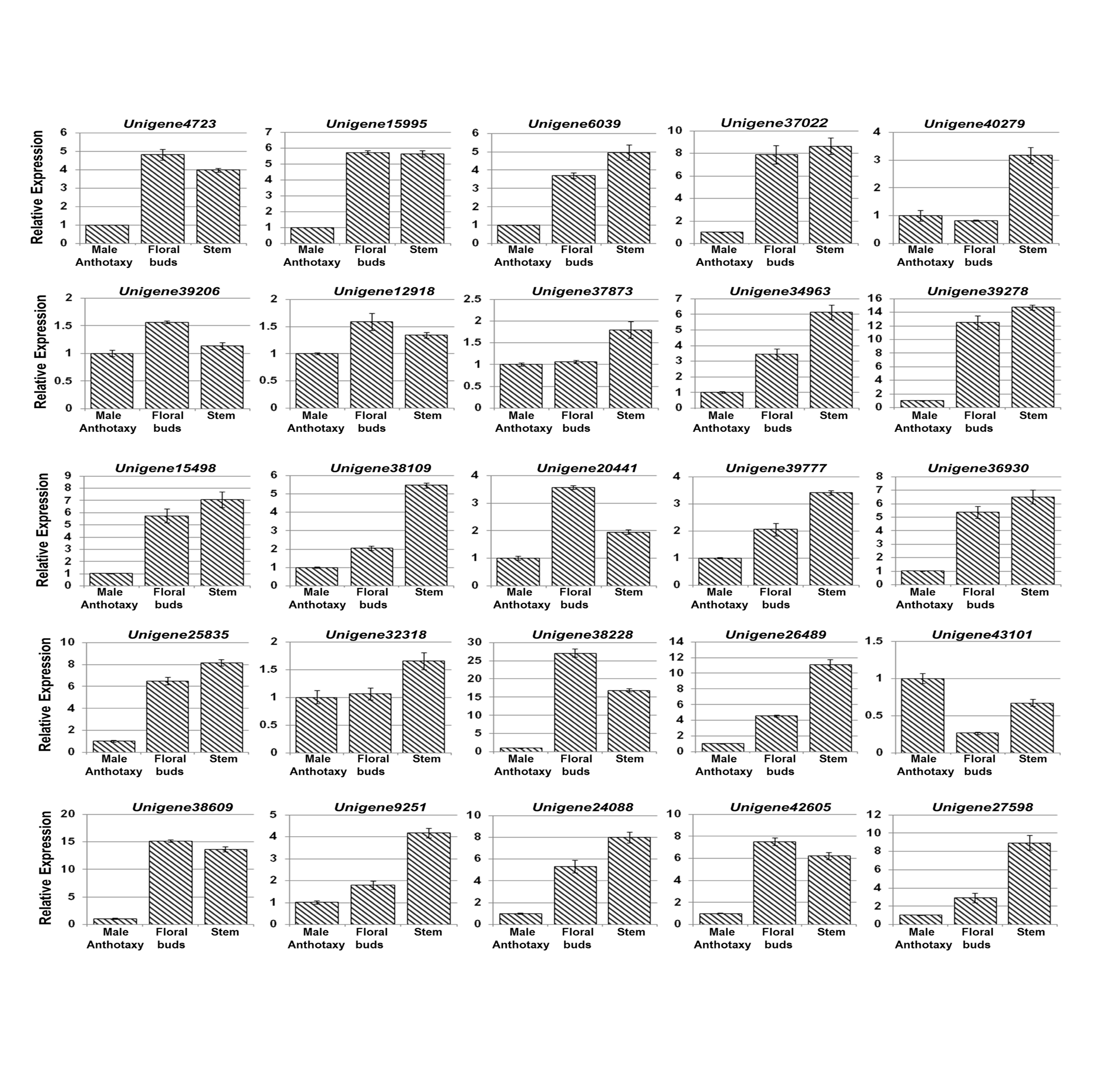

Supplement: S7 Fig — (TIF) [file pone.0135315.s007.tif]

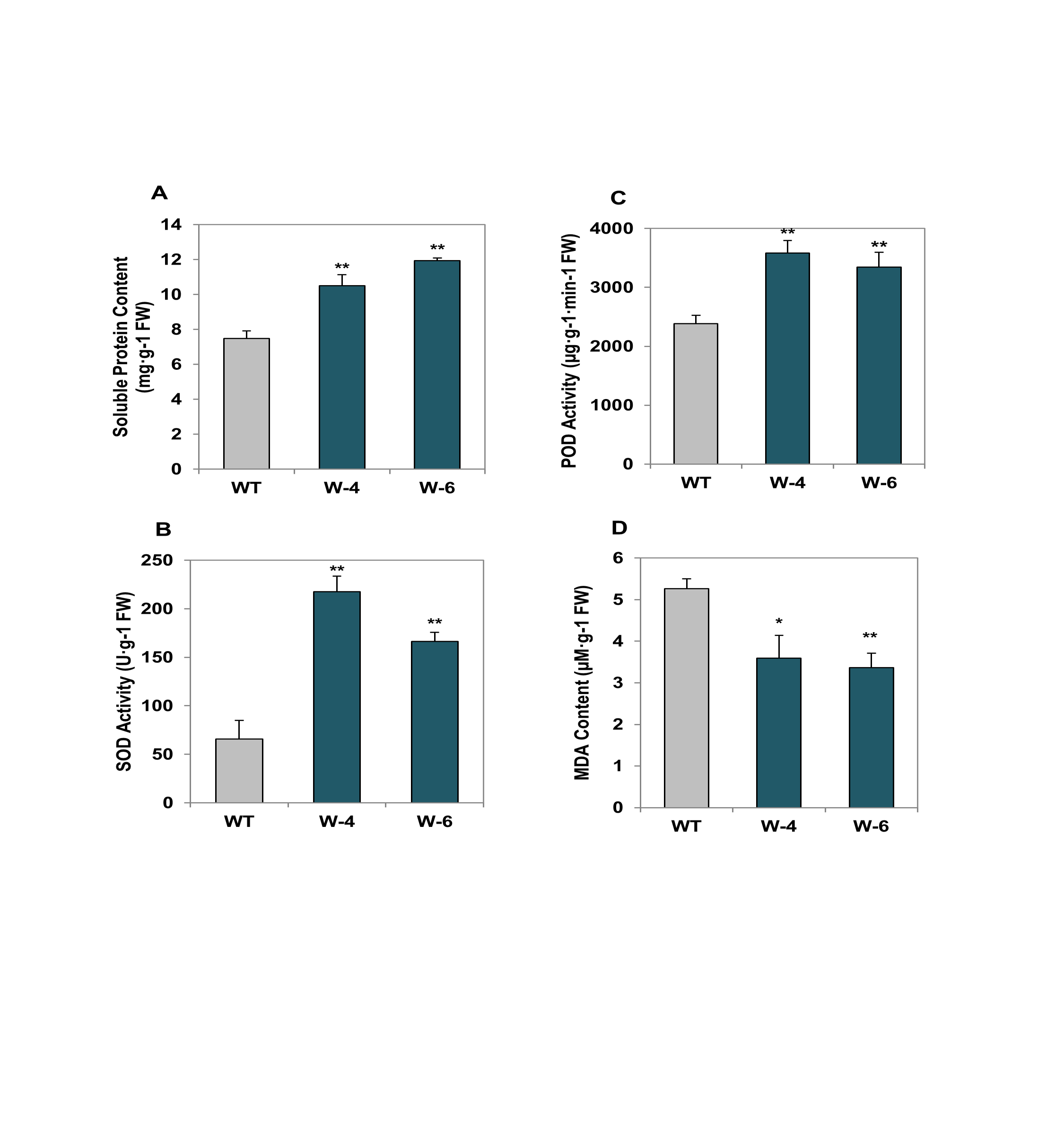

Supplement: S8 Fig — Six-week-old wild type (WT) and T2 transgenic lines (W-4, W-6) were held at 4°C for 24 h. (A) Soluble protein content in WT and T2 transgenic leaves exposed to cold. (B) Superoxide dismutase activity. (C) Peroxidase activity. (D) Malondialdehyde content. The mean values and standard errors were derived from three experimental replicates. (TIF) [file pone.0135315.s008.tif]

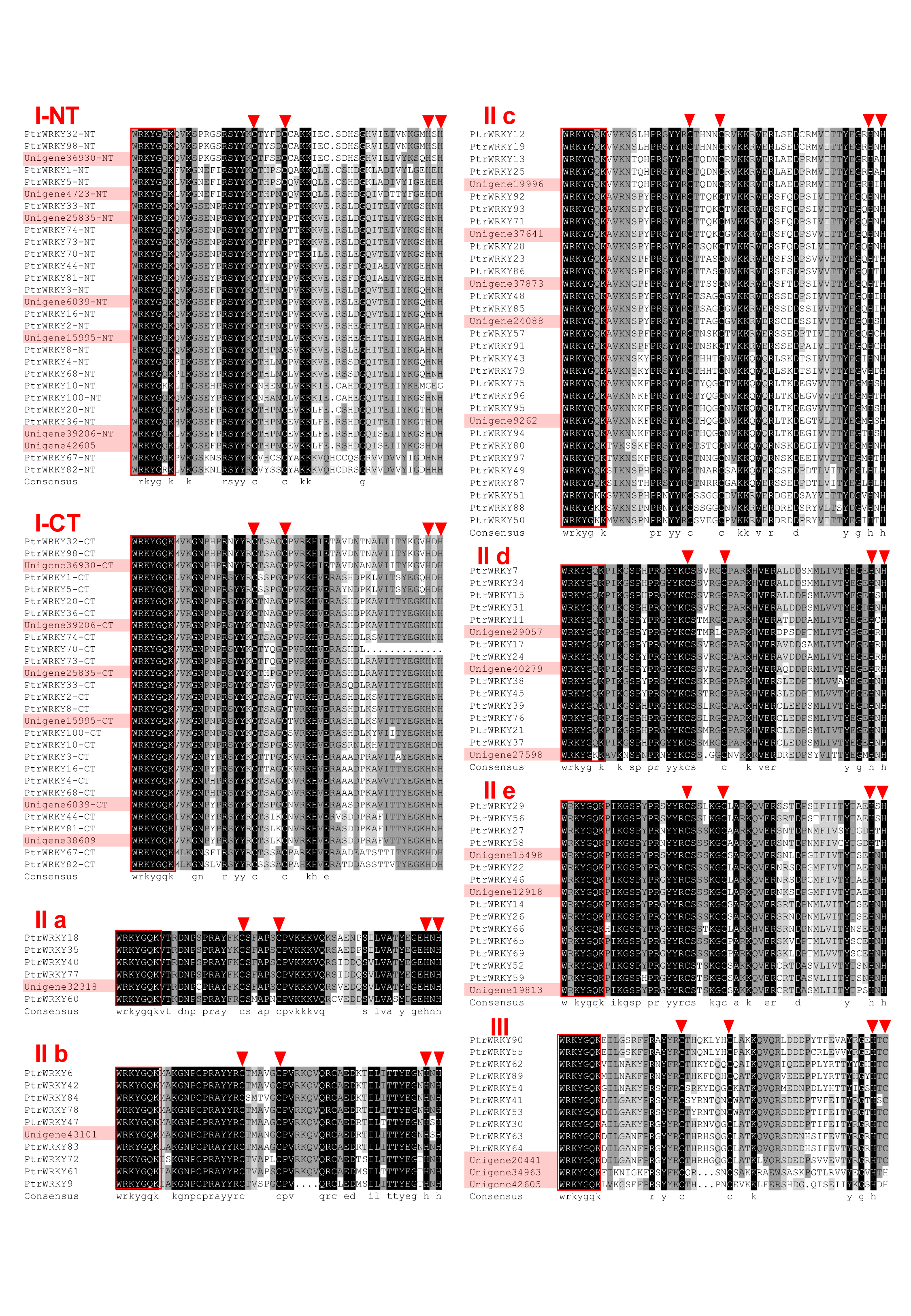

Supplement: S9 Fig — Based on the features of their WRKY domains, the corresponding proteins from C. heterophylla and P. trichocarpa were divided into three groups. Groups II was further classified into five subgroups (IIa, IIb, IIc, IId, IIe). The WRKYGQK domains are indicated with red boxes and the zinc-finger motif sequences are indicated with red triangles. (TIF) [file pone.0135315.s009.tif]
