# Supplementary material for: Expression and Functional Analysis of WRKY Transcription Factors in Chinese Wild Hazel, Corylus heterophylla Fisch
Source: PLoS One. 2015 Aug 13;10(8):e0135315. doi: 10.1371/journal.pone.0135315 (PMC4536078; doi:10.1371/journal.pone.0135315)
Supplement: S1 Table — (DOCX) [file pone.0135315.s010.docx]

**S1 Table.** Primers used for RACE-PCR analysis.

| **Unigene ID** | **Primers sequence** |
| --- | --- |
| Unigene15995 | 3’RACE P1：AAGCAAAATGCCCCCAGCCACTCTT  5’RACE P2：CTGAGGTCCAAGCGGCAGCCTACTC |
| Unigene6039 | 3’RACE P1：GCATCGCAGCCTAGACCAGACAATG  5’RACE P2：CGCATCACCCACTTCCTCGCTATCA |
| Unigene29057 | 3’RACE P1：AAACACGTGGAGAGGGACCCGAGTG  5’RACE P2：ACCCTCCCTTCCTCCCTCCCTTTTG |
| Unigene19996 | 3’RACE P1：GGAAGGTGAGGGAGCCTCGGTTTTG  5’RACE P2：TGAAGCAAAACCGAGGCTCCCTCAC |
| Unigene40279 | 3’RACE P1：CAAGGGCTCACCTTACCCACGAGGA  5’RACE P2：TCCTCGTGGGTAAGGTGAGCCCTTG |
| Unigene37873 | 3’RACE P1：AGCACACGCATCCGAGTCCAGTCAT  5’RACE P2：AACCGGTGTTGCAGAACCGAACCTC |
| Unigene37641 | 3’RACE P1：CTGCTTGAATCTCCACCGTCGATGG  5’RACE P2：CCATCGACGGTGGAGATTCAAGCAG |
| Unigene20441 | 3’RACE P1：TTTGCTCTGGAACAGGGGTGGAAGG  5’RACE P2：TGGCGGAGGTTGGGGCAGAAATTAT |
| Unigene36930 | 3’RACE P1：GTCGGTTGGCGGAGCTGAATTGAAG  5’RACE P2：TGGTTCATTGGCATGCTCCTCCTTG |
| Unigene25835 | 3’RACE P1：TGAACGACAACAATGCAGCCACAGC  5’RACE P2：AATGGCCGATGGCCTAATTGCTGTG |
| Unigene32318 | 3’RACE P1：GAAACCCAGCAGCGCTATTGCCAAG  5’RACE P2：CTCACTTCCGGCGAATCCATTCTGG |
| Unigene42605 | 3’RACE P1：GCCAGCCTCAGGCATTTGTGTCATC  5’RACE P2：TCATCAGACGGCCTGTCAGCAACAA |
| Unigene9262 | 3’RACE P1：GCTTTGTGGGGTCCGAAACTGATGC  5’RACE P2：TGCATCAGTTTCGGACCCCACAAAG |
