# Supplementary material for: Expression and Functional Analysis of WRKY Transcription Factors in Chinese Wild Hazel, Corylus heterophylla Fisch
Source: PLoS One. 2015 Aug 13;10(8):e0135315. doi: 10.1371/journal.pone.0135315 (PMC4536078; doi:10.1371/journal.pone.0135315)
Supplement: S2 Table — (DOCX) [file pone.0135315.s011.docx]

**S2 Table.** Primers used for *Corylus heterophylla* Fisch *WRKYs* expression pattern analysis.

| **Unigene ID** | **Primer Sequence** | **Product length(bp)** | **Purpose** |
| --- | --- | --- | --- |
| *Unigene32318* | F: 5'-CGGGTTCTCGAGGATGCTTC-3' | 119bp | qRT-PCR |
|  | R: 5'-CTCACGCGGTTCATCTCCTC-3' |  | qRT-PCR |
| *Unigene9262* | F: 5'-ATGTCGGAGATGGATGCTCCTGTT-3' | 80bp | qRT-PCR |
|  | R: 5'-TTTGCATCAGTTTCGGACCCCAC-3' |  | qRT-PCR |
| *Unigene37641* | F: 5'-GCCTTCATGACCAAGAGTGAGG-3' | 118bp | qRT-PCR |
|  | R: 5'-GTGTGGTGCATCGATAATAGCTC-3' |  | qRT-PCR |
| *Unigene20441* | F: 5'-CCGTTGATTGGGAGCAAACG-3' | 83bp | qRT-PCR |
|  | R: 5'-GTGGAGATGGTTCAGGAGCTG-3' |  | qRT-PCR |
| *Unigene40279* | F: 5'-GGCCCCCAATCCCTTGAC-3' | 87bp | qRT-PCR |
|  | R: 5'-GCCGGTCCGGTTCAGGAT-3' |  | qRT-PCR |
| *Unigene37873* | F: 5'-CGTTGGATCTTCATCGTTCTCGG-3' | 93bp | qRT-PCR |
|  | R: 5'-CCTAGCGAGCTCTTATCACCTTC-3' |  | qRT-PCR |
| *Unigene15995* | F: 5'-GACCTCCACAGCCAATCAT-3' | 170bp | qRT-PCR |
|  | R: 5'-GTGTATTCAACCTGGGAGCA-3' |  | qRT-PCR |
| *Unigene19996* | F: 5'-GCCTTCACTACCATACCTCC-3' | 96bp | qRT-PCR |
|  | R: 5'-CTTCTCTTTGCTTTGTAGTGG-3' |  | qRT-PCR |
| *Unigene29057* | F: 5'-CTCACTCGCAAACCCTAACC-3' | 145bp | qRT-PCR |
|  | R: 5'-CGAGAAGACGAGGGTCAAAC-3' |  | qRT-PCR |
| *Unigene42605* | F: 5'-CGTGCTCCTCTCCAACAACA-3' | 139bp | qRT-PCR |
|  | R: 5'-CGCTGAAGGTGTTGCTGTTG-3' |  | qRT-PCR |
| *Unigene36930* | F: 5'-CAGAACATCGCATCCAGAGG-3' | 113bp | qRT-PCR |
|  | R: 5'-CAGAGTCGCTTGAGTTCAGTC-3' |  | qRT-PCR |
| *Unigene6039* | F: 5'-GGTGATGCGGAAACTGGAGT-3' | 113bp | qRT-PCR |
|  | R: 5'-GACAGTCCTATGTGAAGCAGC-3' |  | qRT-PCR |
| *Unigene25835* | F: 5'-CAAACTTCACAGCAACAGCC-3' | 145bp | qRT-PCR |
|  | R: 5'-GCCTGAGTGTTGGTCTGTATG-3' |  | qRT-PCR |
| *Unigene39206* | F: 5'-AGAGAGCGATACGGTTAGCC-3' | 90bp | qRT-PCR |
|  | R: 5'-ATGATGCGTCTGCCTATGCT-3' |  | qRT-PCR |
| *Unigene4723* | F: 5'-GCCAGACACCTCATCAAATG-3' | 182bp | qRT-PCR |
|  | R: 5'-CCACTGGAACAGCATCAACA-3' |  | qRT-PCR |
| *Unigene38228* | F: 5'-CCTGGTGGCTATTTATGAAGGG-3' | 100bp | qRT-PCR |
|  | R: 5'-GATATGTGAATAGAAGAACCATGG-3' |  | qRT-PCR |
| *Unigene38609* | F: 5'-GGCAGAAGGTTGTCAAGGGA-3' | 167bp | qRT-PCR |
|  | R: 5'-GATTTGTGCTCCTCACTGGC-3' |  | qRT-PCR |
| *Unigene43101* | F: 5'-AATCACAGCCATCCGTTGC-3' | 109bp | qRT-PCR |
|  | R: 5'-TTCATTAGCCCGTCAGCACT-3' |  | qRT-PCR |
| *Unigene26489* | F: 5'-TTCAACATCTTCGTCGCTCG-3' | 93bp | qRT-PCR |
|  | R: 5'-CCCACTCCATCTCAGAATCAAC-3' |  | qRT-PCR |
| *Unigene34963* | F: 5'-GATTGGTGTTGCCTGAAGATGG-3' | 150bp | qRT-PCR |
|  | R: 5'-GCTTCGACGCCGACCACTCAGC-3' |  | qRT-PCR |
| *Unigene12918* | F: 5'-CCAAAGCACAAACACAGAGAC-3' | 147bp | qRT-PCR |
|  | R: 5'-ATGTCGGAAGAGAGACCCTCA-3' |  | qRT-PCR |
| *Unigene9251* | F: 5'-GCTGATTACACTTCGGGAGGA-3' | 150bp | qRT-PCR |
|  | R: 5'-TCTTGGCGATTCTGCTGCG-3' |  | qRT-PCR |
| *Unigene15498* | F: 5'-AGTCAGCACAAGAGGGTGG-3' | 95bp | qRT-PCR |
|  | R: 5'-TCCTTTGATGGGTTTCTGACCA-3' |  | qRT-PCR |
| *Unigene19813* | F: 5'-AGCCATAACCATCCAGGTCC-3' | 125bp | qRT-PCR |
|  | R: 5'-CTGGT AGCTCTTGCT GTTCCTC-3' |  | qRT-PCR |
| *Unigene24088* | F: 5'-GCAACAGCAACAAACGCTTG-3' | 158bp | qRT-PCR |
|  | R: 5'-TCCTCTTCTCCACCAGCCTT-3' |  | qRT-PCR |
| *Unigene27598* | F: 5'-GTCAAGAACAGCCCAAACCC-3' | 119bp | qRT-PCR |
|  | R: 5'-TCCCTCGTATGTCGTTATCACG-3' |  | qRT-PCR |
| *Unigene37022* | F: 5'-CCTTCTTTGCCCATTTCTCCG-3' | 131bp | qRT-PCR |
|  | R: 5'-CGTTGGAGAAGGCAGAATGTTA-3' |  | qRT-PCR |
| *Unigene38109* | F: 5'-GCAGAAGCCACGATGAGGAA-3' | 177bp | qRT-PCR |
|  | R: 5'-GAACTTGTTTGCGGACTGGAC-3' |  | qRT-PCR |
| *Unigene39278* | F: 5'-CGTCATCCTCCAGTTGTTCG-3' | 170bp | qRT-PCR |
|  | R: 5'-CTGTGGGCTTCTCTTTCTCTTG-3' |  | qRT-PCR |
| *Unigene39777* | F: 5'-GCCTGGTTTGGATGTCAACA-3' | 115bp | qRT-PCR |
|  | R: 5'-CACTCTTTGCTCGCTTGTCTT-3' |  | qRT-PCR |
| *Actin* | F: 5'-TGGTCAAGGCTGGGTTTGC-3' | 101bp | qRT-PCR |
|  | R: 5'-CTGACCCATCCCAACCATGA-3' |  | qRT-PCR |
