# Supplementary material for: Expression and Functional Analysis of WRKY Transcription Factors in Chinese Wild Hazel, Corylus heterophylla Fisch
Source: PLoS One. 2015 Aug 13;10(8):e0135315. doi: 10.1371/journal.pone.0135315 (PMC4536078; doi:10.1371/journal.pone.0135315)
Supplement: S3 Table — (DOCX) [file pone.0135315.s012.docx]

**S3 Table.** Characteristics of *WRKY* genes in *Corylus heterophylla* Fisch.

| **Unigene ID** | **Arabidopsis ortholog** | **E-value** |
| --- | --- | --- |
| Unigene39206 | AtWRKY20 | 7.00E-159 |
| Unigene4723 | AtWRKY1 | 3.00E-62 |
| Unigene38228 | AtWRKY40 | 2.00E-23 |
| Unigene38609 | AtWRKY44 | 8.00E-40 |
| Unigene43101 | AtWRKY42 | 3.00E-135 |
| Unigene26489 | AtWRKY41 | 9.00E-06 |
| Unigene34963 | AtWRKY23 | 3.00E-17 |
| Unigene12918 | AtWRKY22 | 3.00E-50 |
| Unigene9251 | AtWRKY47 | 3.00E-11 |
| Unigene15498 | AtWRKY27 | 2.00E-39 |
| Unigene19813 | AtWRKY35 | 2.00E-35 |
| Unigene24088 | AtWRKY48 | 6.00E-60 |
| Unigene27598 | AtWRKY51 | 5.00E-35 |
| Unigene37022 | AtWRKY26 | 8.00E-13 |
| Unigene38109 | AtWRKY6 | 6.00E-67 |
| Unigene39278 | AtWRKY15 | 6.00E-20 |
| Unigene39777 | AtWRKY31 | 4.00E-34 |
