# Supplementary material for: Expression and Functional Analysis of WRKY Transcription Factors in Chinese Wild Hazel, Corylus heterophylla Fisch
Source: PLoS One. 2015 Aug 13;10(8):e0135315. doi: 10.1371/journal.pone.0135315 (PMC4536078; doi:10.1371/journal.pone.0135315)
Supplement: S4 Table — (DOCX) [file pone.0135315.s013.docx]

**S4 Table.** *WRKY* expression patterns under abiotic stress.

| **Unigene ID** | **Cold stress** | **Drought stress** | **Salinity stress** |
| --- | --- | --- | --- |
| Unigene15995 | **/** | **/** | **/** |
| Unigene6039 | **+** | **+** | **+** |
| Unigene29057 | **+** | **+** | **+** |
| Unigene19996 | **+** | **+** | **+** |
| Unigene40279 | **+** | **+** | **+** |
| Unigene37873 | **+** | **+** | **+** |
| Unigene37641 | **+** | **+** | **+** |
| Unigene20441 | **+** | **+** | **+** |
| Unigene36930 | **/** | **+** | **+** |
| Unigene25835 | **+** | **+** | **+** |
| Unigene32318 | **+** | **+** | **+** |
| Unigene42605 | **+** | **+** | **+** |
| Unigene9262 | **+** | **+** | **+** |
| Unigene39206 | / | **+** | **+** |
| Unigene4723 | **+** | **+** | **+** |
| Unigene37022 | **+** | **+** | **+** |
| Unigene12918 | **+** | **+** | **+** |
| Unigene34963 | - | **+** | **+** |
| Unigene39278 | **+** | **+** | **+** |
| Unigene15498 | **+** | **+** | **+** |
| Unigene38109 | **+** | **+** | **+** |
| Unigene39777 | **+** | **+** | **+** |
| Unigene19813 | **+** | **+** | **+** |
| Unigene38228 | **+** | **+** | **+** |
| Unigene26489 | **+** | **+** | **+** |
| Unigene43101 | **+** | **+** | **+** |
| Unigene38609 | **+** | **/** | **+** |
| Unigene9251 | **+** | **+** | **+** |
| Unigene24088 | - | **+** | **-** |
| Unigene27598 | **/** | **+** | **+** |

Gene expression was evaluated using qRT-PCR and repeated at least three times with

independent biological samples as starting material. The results shown were consistent

in all three replicates. ‘+’ and ‘-’ correspond to up-regulated and down-regulated,

respectively, by various stresses and ‘/’ indicates that the expression level was not

altered by the corresponding stress treatment.
