# Supplementary material for: Expression and Functional Analysis of WRKY Transcription Factors in Chinese Wild Hazel, Corylus heterophylla Fisch
Source: PLoS One. 2015 Aug 13;10(8):e0135315. doi: 10.1371/journal.pone.0135315 (PMC4536078; doi:10.1371/journal.pone.0135315)
Supplement: S5 Table — (DOCX) [file pone.0135315.s014.docx]

**S5 Table.** Subcellular localization prediction using Euk-mPLoc.

| **Protein ID** | **Prediction results** |
| --- | --- |
| Unigene15995 | Nucleus |
| Unigene6039 | Nucleus |
| Unigene29057 | Nucleus |
| Unigene19996 | Nucleus |
| Unigene40279 | Nucleus |
| Unigene37873 | Nucleus |
| Unigene37641 | Nucleus |
| Unigene20441 | Nucleus |
| Unigene36930 | Nucleus |
| Unigene25835 | Nucleus |
| Unigene32318 | Nucleus |
| Unigene42605 | Nucleus |
| Unigene9262 | Nucleus |
| Unigene39206 | Nucleus |
| Unigene4723 | Nucleus |
| Unigene37022 | Cytoplasm |
| Unigene12918 | Nucleus |
| Unigene34963 | Nucleus |
| Unigene39278 | Nucleus |
| Unigene15498 | Nucleus |
| Unigene38109 | Nucleus |
| Unigene39777 | Nucleus |
| Unigene19813 | Nucleus |
| Unigene38228 | Nucleus |
| Unigene26489 | Nucleus |
| Unigene43101 | Nucleus |
| Unigene38609 | Nucleus |
| Unigene9251 | Nucleus |
| Unigene24088 | Nucleus |
| Unigene27598 | Nucleus |
